# Non-Canonical Role of PDK1 as a Negative Regulator of Apoptosis through Macromolecular Complexes Assembly at the ER–Mitochondria Interface in Oncogene-Driven NSCLC

**DOI:** 10.3390/cancers13164133

**Published:** 2021-08-17

**Authors:** Viviana De Rosa, Francesca Iommelli, Cristina Terlizzi, Eleonora Leggiero, Rosa Camerlingo, Giovanna G. Altobelli, Rosa Fonti, Lucio Pastore, Silvana Del Vecchio

**Affiliations:** 1 Institute of Biostructures and Bioimaging, National Research Council, 80145 Naples, Italy; viviana.derosa@ibb.cnr.it (V.D.R.); francesca.iommelli@ibb.cnr.it (F.I.); rosa.fonti@ibb.cnr.it (R.F.); 2 Department of Advanced Biomedical Sciences, University of Naples “Federico II”, 80131 Naples, Italy; cristina.terlizzi@unina.it (C.T.); ggaltobe@unina.it (G.G.A.); 3 CEINGE-Biotecnologie Avanzate, 80131 Naples, Italy; leggiero@ceinge.unina.it (E.L.); lucio.pastore@unina.it (L.P.); 4 Department of Cell Biology and Biotherapy, Istituto Nazionale Tumori-IRCCS-Fondazione G. Pascale, 80131 Naples, Italy; r.camerlingo@istitutotumori.na.it; 5 Department of Molecular Medicine and Medical Biotechnology, University of Naples “Federico II”, 80131 Naples, Italy

**Keywords:** PDK1, OXPHOS, glycolysis, apoptosis, drug resistance

## Abstract

**Simple Summary:**

Co-targeting of glucose metabolism and oncogene drivers in patients with non-small cell lung cancer (NSCLC) has been proposed as a potentially effective therapeutic strategy. Here, we demonstrate that downregulation of pyruvate dehydrogenase kinase 1 (PDK1), an enzyme of glycolytic cascade, enhances maximal respiration of cancer cells by upregulating mitochondrial complexes of oxidative phosphorylation (OXPHOS) and improves tumor response to tyrosine kinase inhibitors by promoting apoptosis. Furthermore, we provided consistent evidence that PDK1 drives the formation of macromolecular complexes at the ER–mitochondria interface involving PKM2, Bcl-2 and Bcl-xL and serves as an indirect anchorage of anti-apoptotic proteins to the mitochondrial membrane. Our findings taken together highlighted a non-canonical role of PDK1 as a negative regulator of apoptosis, thus coupling the glycolytic phenotype to drug resistance. The major translational relevance of this study is to provide a rational basis for combined therapeutic strategies targeting PDK1 and oncogene drivers in NSCLC patients.

**Abstract:**

Here, we tested whether co-targeting of glucose metabolism and oncogene drivers may enhance tumor response to tyrosine kinase inhibitors (TKIs) in NSCLC. To this end, pyruvate dehydrogenase kinase 1 (PDK1) was stably downregulated in oncogene-driven NSCLC cell lines exposed or not to TKIs. H1993 and H1975 cells were stably transfected with scrambled (shCTRL) or PDK1-targeted (shPDK1) shRNA and then treated with MET inhibitor crizotinib (1 µM), double mutant EGFR^L858R/T790M^ inhibitor WZ4002 (1 µM) or vehicle for 48 h. The effects of PDK1 knockdown on glucose metabolism and apoptosis were evaluated in untreated and TKI-treated cells. PDK1 knockdown alone did not cause significant changes in glycolytic cascade, ATP production and glucose consumption, but it enhanced maximal respiration in shPDK1 cells when compared to controls. When combined with TKI treatment, PDK1 downregulation caused a strong enhancement of OXPHOS and a marked reduction in key glycolytic enzymes. Furthermore, increased levels of apoptotic markers were found in shPDK1 cells as compared to shCTRL cells after treatment with TKIs. Co-immunoprecipitation studies showed that PDK1 interacts with PKM2, Bcl-2 and Bcl-xL, forming macromolecular complexes at the ER–mitochondria interface. Our findings showed that downregulation of PDK1 is able to potentiate the effects of TKIs through the disruption of macromolecular complexes involving PKM2, Bcl-2 and Bcl-xL.

## 1. Introduction

The main source of energy in cancer cells is aerobic glycolysis that also provides metabolic intermediates for biosynthetic pathways of macromolecules required for tumor cell growth, proliferation and survival [1]. The activation of oncogenes or loss of function of tumor suppressor genes drives neoplastic transformation and causes a metabolic reprogramming characterized by a shift from mitochondrial oxidative phosphorylation (OXPHOS) to aerobic glycolysis [2]. The acquired glycolytic phenotype is maintained and regulated by a complex signaling network orchestrated by the oncogene driver and highly dependent on cellular context and microenvironmental conditions [3]. Aberrant signaling through PI3K/AKT pathway, the activation status of two opposing metabolic sensors such as mTOR and AMPK, the transcriptional activity of HIF-1, MYC and p53 are all considered the main regulators of glucose metabolism in cancer cells [3,4]. An enhanced flux of glucose through glycolysis is maintained by AKT-dependent upregulation of hexokinase II and phosphofructokinase [5], whereas the glycolytic cascade is slowed down by overexpression of low-activity dimeric form of PKM2 in cancer cells [6,7]. Diversion of pyruvate flux from mitochondrial oxidative phosphorylation toward conversion to lactate is mainly achieved by a complex regulation of pyruvate dehydrogenase kinase (PDK) [8].

The family of PDK enzymes includes four isoforms (PDK1-PDK4) located in the mitochondrial matrix where they can phosphorylate and inactivate the pyruvate dehydrogenase complex (PDHC), a multi-enzyme complex, that catalyzes the conversion of pyruvate to acetyl-CoA. PDK phosphorylation, occurring at any one site of three serine residues (Ser293, Ser300 and Ser232) on the alpha subunit (E1α) of the E1 component, can inactivate the whole PDHC [9]. Among the four isoforms, PDK1 has been extensively studied in cancer and its upregulation has been reported in a variety of tumors including head and neck cancer and breast and lung cancer [10]. PDK1 is a direct target of HIF-1α in cancer cells under both hypoxic and normoxic conditions [11,12], and its downregulation by short hairpin RNA reverts the glycolytic phenotype, inhibits tumor growth and reduces invasiveness in several tumor cell lines [13]. Similarly, inhibition of PDK by dichloroacetate (DCA) in cancer cells shifts glucose metabolism from glycolysis to oxidative phosphorylation and increases ROS production with subsequent cell death [14]. Pan-inhibition of PDKs was also reported to enhance the effects of cytotoxic drugs such as cisplatin and doxorubicin [15,16,17]. Moreover, the inhibition of PDK1 was reported to enhance the anticancer effects of EGFR tyrosine kinase inhibitors (TKIs) in lung cancer [18,19]. However, the mechanism by which inhibition of PDK1 causes cell death and generates a synergistic effect with anticancer drugs is not completely elucidated.

In oncogene-driven non-small cell lung cancer (NSCLC) we have previously shown that inhibition of the oncogene driver by TKIs causes a metabolic switch from glycolysis to oxidative phosphorylation through the concerted downregulation of HKII and p-PKM2^Tyr105^ and upregulation of OXPHOS [20,21]. Similar results were obtained in Bcr-Abl-driven chronic myelogenous leukemia cells [22]. OXPHOS upregulation was associated with an increased intracellular ATP production and an enhanced oxygen consumption rate supporting the notion that the mitochondria of cancer cells are functional. Here, we tested whether knocking down PDK1 by short hairpin RNA may enhance the effects of TKIs on mitochondrial oxidative phosphorylation and improve tumor response to these targeted agents in NSCLC. Furthermore, we investigated the molecular mechanisms triggered by the absence of PDK1 that eventually potentiate the effects of TKIs.

## 2. Results

### 2.1. Silencing of PDK1 by Targeted shRNA and Effects on Glucose Metabolism

**Figure 1:**
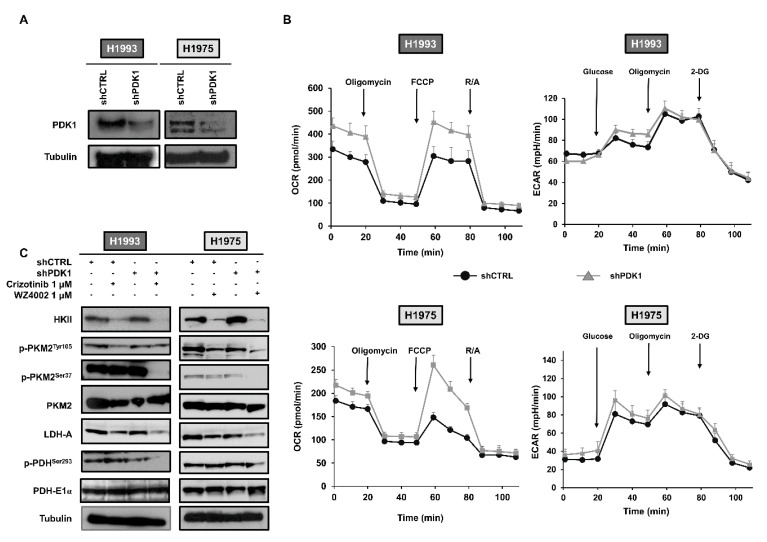
Effects of PDK1 downregulation on oxygen consumption rate and glycolytic cascade. (**A**) H1993 and H1975 cells were stably transfected with PDK1 targeted (shPDK1) and non-targeting (shCTRL) short hairpin RNA. Representative Western blotting showed downregulation of PDK1 protein. (**B**) OCR and ECAR analyses were performed in shCTRL and shPDK1 H1993 and H1975 transfected cells. Error bars represent SD. (**C**) Representative Western blotting of glycolytic cascade obtained from whole cell lysates of shCTRL and shPDK1 H1993 and H1975 cells in basal conditions and after treatment with crizotinib or WZ4002, respectively. Tubulin served to ensure equal loading. The uncropped Western Blot images can be found in Figures S4–S6.

In order to test whether the downregulation of PDK1 may potentiate the effects of MET and EGFR inhibitors, we stably transfected H1993 and H1975 cells with a lentiviral vector expressing either a PDK1 targeted shRNA (shPDK1) and non-targeting shRNA (shCTRL). Figure 1A shows the reduction in PDK1 levels in both cell lines after transfection with shPDK1 as compared to shCTRL transfected cells. In order to evaluate the effects of PDK1 downregulation on glucose metabolism, we performed OCR and ECAR analysis on transfected H1993 and H1975 cells. Figure 1B shows that PDK1 knockdown causes an enhancement of maximal respiration in both cell lines along with a mild OCR increase in basal conditions. Conversely, no significant changes of ECAR were observed in response to PDK1 downregulation in both cell lines (Figure 1B). In parallel experiments, we tested whether PDK1 downregulation enhances the contribution of glucose to TCA by measuring citrate levels, the first TCA intermediate. Figure S1 shows the statistically significant increase in citrate levels in shPDK1 cells as compared to shCTRL cells.

The levels of key glycolytic proteins were tested in shCTRL and shPDK1 H1993 and H1975 cells in basal conditions and in response to 48 h treatment with crizotinib or WZ4002 (1 μM). Figure 1C showed that treatment with crizotinib or WZ4002 caused, as expected, a reduction in HKII, p-PKM2^Tyr105^ and LDH-A levels in shCTRL H1993 and H1975 cells. In both cell lines, downregulation of PDK1 caused a slight decrease in p-PKM2^Tyr105^ and LDH-A levels as compared to shCTRL cells. Treatment with crizotinib and WZ4002 in PDK1 silenced cells caused an enhanced reduction in all selected glycolytic proteins as compared to the effects of the same treatment in shCTRL cells, except for PKM2 and PDH-E1α.

**Figure 2:**
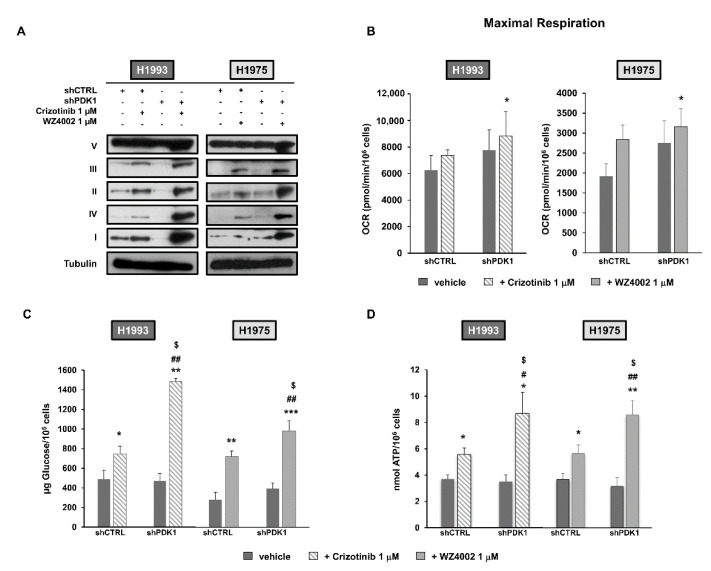
Combined effects of PDK1 knockdown and TKI treatment on OXPHOS. (**A**) Representative Western blotting of OXPHOS in whole cell lysates of shCTRL and shPDK1 H1993 and H1975 cells in basal conditions and after treatment with crizotinib or WZ4002, respectively. Tubulin served to ensure equal loading. (**B**) OCR measurements were obtained after FCCP stimulation in order to evaluate maximal respiration in shCTRL and shPDK1 H1993 and H1975 cells treated or not with crizotinib or WZ4002, respectively. Data are normalized for 10^6^ cells and expressed as mean ± SD. The symbol * indicates statistically significant differences (*p* < 0.05) versus treated shCTRL cells. (**C**,**D**) Concentration of residual glucose in conditioned media (**C**) and intracellular ATP levels (**D**) of untreated and treated cells. At least three independent assays were performed. Data are normalized for 10^6^ cells and expressed as mean ± SE. The symbol * indicates statistically significant differences versus untreated shCTRL cells with * *p* < 0.05, ** *p* < 0.01 and *** *p* < 0.001; the symbol # indicates statistically significant differences versus untreated shPDK1 cells with # *p* < 0.05 and ## *p* < 0.01; the symbol $ indicates statistically significant differences versus treated shCTRL cells with $ *p* < 0.05. The uncropped Western Blot images can be found in Figure S7.

Considering oxidative phosphorylation, an upregulation of mitochondrial subunits (I–IV) was observed in shCTRL cells after treatment with crizotinib and WZ4002 (Figure 2A). Downregulation of PDK1 in combination with TKI treatment caused a strong enhancement of OXPHOS in both cell lines that was much higher than that observed in treated shCTRL cells. Similar results were obtained with targeted PDK1 siRNA, in the presence or absence of TKIs treatment (Figure S2).

In agreement with this finding, maximal respiration of shPDK1 cells treated with crizotinib or WZ4002 showed a modest increase, although statistically significant, as compared to that observed in shCTRL cells exposed to the same inhibitors (Figure 2B). Then, the effects of PDK1 downregulation on extracellular glucose consumption and intracellular ATP production were evaluated in untreated and treated cells. As shown in Figure 2C, PDK1 silencing combined with TKI treatment caused a significant increase in residual glucose in the conditioned media of both cell lines that was significantly higher than that obtained in shCTRL cells exposed to the same TKI. A parallel significant increase in intracellular ATP levels was observed in shPDK1 cells as compared to shCTRL cells, both exposed to crizotinib or WZ40402 (Figure 2D). These findings taken together indicate that downregulation of PDK1 potentiates the effects of TKI treatment on oxidative phosphorylation by enhancing the oxygen consumption and ATP production.

### 2.2. Effects of PDK1 Knockdown on Apoptotic Response to TKI Treatment

**Figure 3:**
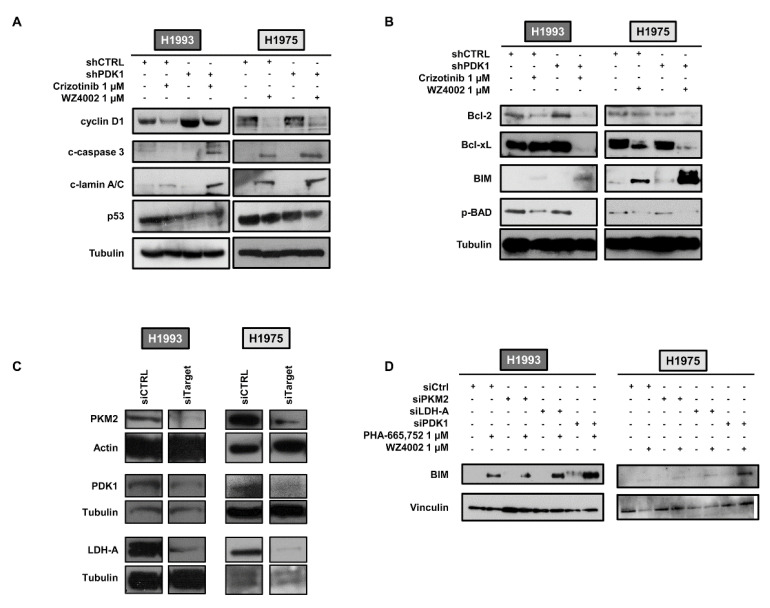
Combined effects of PDK1 knockdown and TKI treatment on cyclin D1, apoptotic markers and levels of Bcl-2 family members. (**A,B**) Western blot analysis of whole cell lysates from shCTRL and shPDK1 H1993 and H1975 cells in basal conditions and after treatment with crizotinib or WZ4002, respectively, showing levels of cyclin D1, cleaved caspase 3, cleaved lamin a/c and p53 (**A**) along with levels of Bcl-2 family members (**B**). (**C**) LDH-A, PKM2 or PDK1 silencing in H1993 and H1975 cells by siRNA and levels of the target protein. (**D**) Representative Western blots showing BIM levels in siRNA transfected H1993 and H1975 cells in basal conditions and after treatment with TKIs. Tubulin, actin or vinculin served to ensure equal loading. The uncropped Western Blot images can be found in Figures S8–S14.

In order to test whether PDK1 silencing may also potentiate the effects of TKI treatment on tumor growth, we investigated the expression levels of several key proteins of proliferation and apoptosis. Figure 3A showed that cyclin D1 levels were equally reduced in response to TKIs in shCTRL and shPDK1 cells. Conversely a strong enhancement of the apoptotic cascade was observed in shPDK1 cells treated with TKIs compared to shCTRL cells subjected to the same treatment. When the levels of Bcl-2 family proteins were determined, we found that knockdown of PDK1 alone did not cause any change in anti-apoptotic Bcl-2 and Bcl-xL levels as well as in pro-apoptotic BIM and p-BAD. When shPDK1 cells were exposed to treatment with TKIs, a strong reduction in Bcl-xL and p-BAD was observed along with an enhanced upregulation of BIM as compared to similarly treated shCTRL cells (Figure 3B). A slight reduction in Bcl-2 levels was also observed in treated shPDK1 cells as compared to treated shCTRL cells. Furthermore, Annexin V assays in untreated and treated shCTRL and shPDK1 cells showed a progressive increase in apoptosis as compared to untreated shCTRL cells already achieving statistical significance in untreated shPDK1 (Figure S3).

In order to test whether the enhancement of apoptotic cascade induced by TKIs was not a generic stress response to the downregulation of glycolysis, we silenced glycolytic enzymes immediately upstream and downstream of PDK1 and evaluated the effects of TKI treatment on BIM upregulation. Figure 3C,D shows that knockdown of PKM2 and LDH-A by targeted siRNA did not cause significant changes of BIM levels upon exposure to TKIs whereas PDK1 silencing by targeted siRNA caused a strong enhancement of BIM upregulation after treatment than compared to treated siCTRL cells. Taken together, these findings indicate that PDK1 may modulate drug-induced apoptosis through an unknown mechanism potentially involving mitochondria and endoplasmic reticulum (ER).

### 2.3. PDK1-Dependent Protein–Protein Interactions at ER–Mitochondria Interface

**Figure 4:**
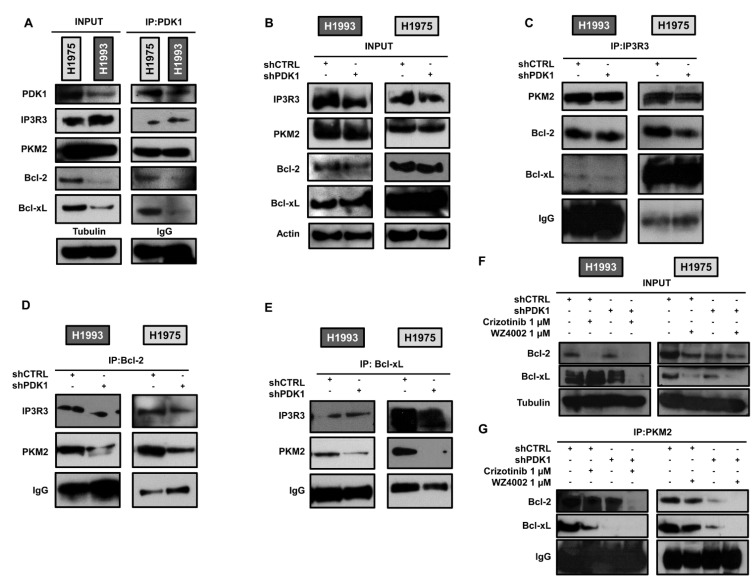
PDK1-dependent protein–protein interactions by immunoprecipitation assays. (**A**) Direct or indirect interactions of PDK1 and selected proteins at the ER–mitochondria interface in H1993 and H1975 parental cell lines. (**B**) Levels of indicated proteins in whole cell lysates of shCTRL and shPDK1 H1993 and H1975 cells. (**C**) Immunoprecipitation with anti-IP3R3 antibody and blotting for the indicated proteins. (**D**) Immunoprecipitation with anti-Bcl-2 antibody and blotting for the indicated proteins. (**E**) Immunoprecipitation with anti-Bcl-xL antibody and blotting for the indicated proteins. (**F**) Whole lysates of untreated and treated shCTRL and shPDK1 H1993 and H1975 cells subjected to (**G**) immunoprecipitation with anti-PKM2 antibody followed by blotting for the indicated proteins. Tubulin and actin served to ensure equal loading. The uncropped Western Blot images can be found in Figures S15–S22.

In order to elucidate the mechanism by which PDK1 downregulation enhances the apoptotic cascade, we evaluated PDK1-dependent protein–protein interaction at the ER–mitochondria interface. Immunoprecipitation of whole cell lysates of parental H1993 and H1975 cells with anti-PDK1 antibody showed that PDK1 directly or indirectly interacts with IP3R3, PKM2, Bcl-2 and Bcl-xL (Figure 4A).

Then, we performed immunoprecipitation with anti-IP3R3 antibodies in both transfected cell lines and no significant changes were found in the interactions between IP3R3 and PKM2 or Bcl-2/Bcl-xL in the presence or absence of PDK1 (Figure 4B,C). Conversely, immunoprecipitation with anti-Bcl-2 and anti-Bcl-xL antibodies showed that knockdown of PDK1 caused a strong reduction in the interaction between Bcl-2/Bcl-xL and PKM2 (Figure 4B,D,E) indicating that PDK1 has a crucial role in promoting these interactions. Immunoprecipitation of whole cell lysates with anti-PKM2 antibodies not only confirmed the reduction in interaction of PKM2 with Bcl-2/Bcl-xL in the absence of PDK1 but showed that treatment with TKIs abolished this interaction in shPDK1 cells (Figure 4F,G). These findings suggest that the absence of PDK1 may cause a redistribution of Bcl-2/Bcl-xL in different cellular compartments, thus favouring the action of pro-apoptotic members of the same family.

**Figure 5:**
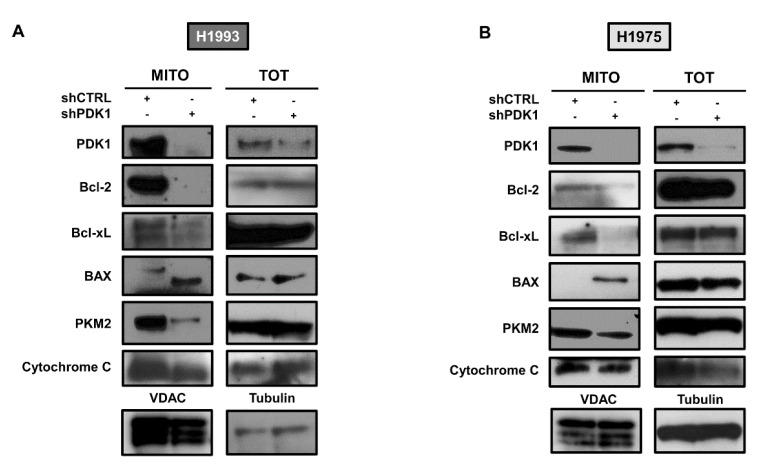
PDK1-dependent localization of selected proteins in mitochondrial fractions. (**A,B**) Levels of PDK1, Bcl-2, Bcl-xL, BAX, PKM2 and cytochrome C in mitochondrial fractions and whole cell lysates of shCTRL and shPDK1 H1993 (**A**) and H1975 (**B**) cells. VDAC and tubulin served to ensure equal loading. The uncropped Western Blot images can be found in Figures S23 and S24.

In order to test the mitochondrial localization of the selected proteins, we evaluated their levels in mitochondrial fractions and whole cell lysates. In both cell lines, knockdown of PDK1 caused a strong reduction in Bcl-2, Bcl-xL and PKM2 levels in mitochondria along with an increase in Bax levels (Figure 5A,B). These findings taken together suggest that PDK1, in addition to its canonical role as a glycolytic enzyme, may serve to anchor PKM2 and anti-apoptotic proteins such as Bcl-2/Bcl-xL to the mitochondrial membrane and, thus, indirectly affecting apoptotic cascade.

### 2.4. Effects of PDK1 Knockdown on Tumor Growth in Animal Models

**Figure 6:**
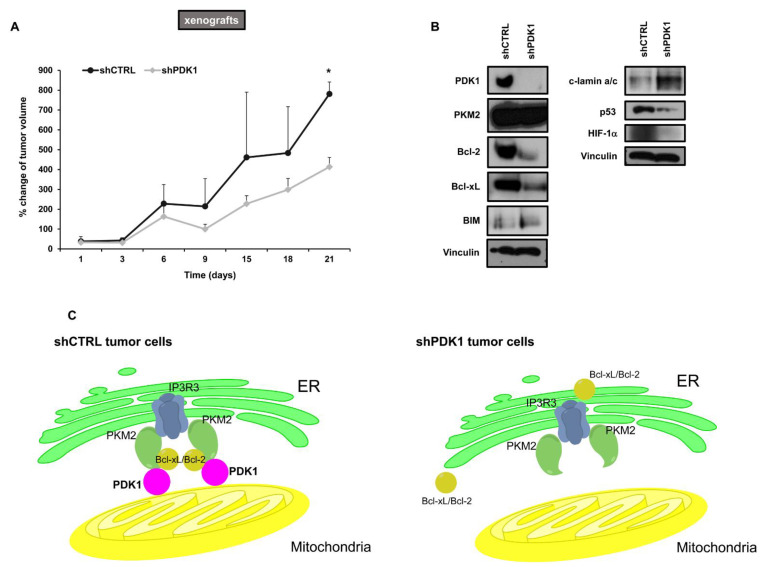
Effects of PDK1 knockdown on tumor growth and levels of selected proteins in surgically excised tumors. (**A**) Tumor volume of H1993 shCTRL and shPDK1 xenografts measured over time. The symbol * indicates statistically significant difference versus shCTRL at 21 days with * *p* < 0.05. (**B**) Western blot analysis of whole lysates from surgically excised xenografts showing levels of Bcl-2 family proteins, p53 and HIF-1α. Vinculin served to ensure equal loading. (**C**) Schematic representation of the PDK1-dependent assembly of macromolecular complexes involving PKM2 and Bcl-2/Bcl-xL at the ER–mitochondria interface. The uncropped Western Blot images can be found in Figure S25.

Nude mice were subcutaneously injected with H1993 cells silenced with either shCTRL or shPDK1 and tumor volumes were monitored over time. At day 21, tumor volumes of shCTRL-silenced H1993 xenografts were significantly higher than those achieved by shPDK1-silenced H1993 xenografts indicating that downregulation of PDK1 reduces tumor growth (Figure 6A). Then, we evaluated the levels of the selected proteins in H1993 shCTRL and shPDK1 xenografts. Figure 6B shows that downregulation of PDK1 caused a reduction in Bcl-2 and Bcl-xL whereas BIM and c-lamin a/c levels increased as compared to the control tumors, thus confirming that PDK1 enhances the sensitivity to apoptotic stimuli. In fact, the different growth conditions of xenografts as compared to cultured tumor cells may induce an additional stress accounting for the enhanced apoptotic response in shPDK1 xenografts compared to the shCTRL tumor.

## 3. Discussion

Our study showed that downregulation of PDK1 combined with TKI treatment strongly enhances oxidative phosphorylation through the upregulation of OXPHOS and reduction in glycolysis, resulting in an increase in ATP production and reduced glucose external supply in oncogene-driven NSCLC cell lines. Silencing of PDK1 alone did not cause any significant change in glucose metabolism, indicating that diversion of pyruvate from glycolysis toward oxidative phosphorylation in oncogene-driven tumors requires intervention at additional levels in glycolysis. Furthermore, the effects of TKI treatment on glycolysis, OXPHOS and apoptosis were strongly potentiated by PDK1 knockdown. Notably, our study highlighted the formation of large molecular complexes at the ER–mitochondria interface involving PDK1, PKM2, Bcl-2/Bcl-xL and IP3R3 in which PDK1 serves as an indirect anchorage of anti-apoptotic proteins to the mitochondrial membrane. In the absence of PDK1, such complexes were disrupted and the levels of Bcl-2/Bcl-xL were reduced in mitochondrial fractions, thus promoting apoptosis.

Previous studies reported that the silencing of PDK1 or DCA treatment causes a metabolic shift from glycolysis to OXPHOS in different types of cancer including head and neck cancer [13], clear-cell renal cell carcinoma [23] and breast cancer [24]. Our findings are in agreement with these results since PDK1 knockdown enhances maximal respiration in oncogene-driven NSCLC cells. However, downregulation of PDK1 alone does not permit the inhibition of the glycolytic cascade when it is under the oncogene driver control. As previously reported, inhibition of the oncogene driver by treatment with TKI alone downregulates glycolysis and reactivates oxidative phosphorylation [20]. In fact, TKI treatment causes the reduction in HKII, p-PKM2^Tyr105^ and LDH-A with the subsequent decrease in pyruvate and lactate levels. However, despite the reduction in pyruvate, its entrance in TCA cycle is enhanced by low levels of p-PKM2^Tyr105^, favoring the tetrameric form of this enzyme and by the higher activity of PDHC caused by PDK1 downregulation. Moreover, combining PDK1 knockdown with TKI treatment further enhanced oxidative phosphorylation and ATP production.

The pan-specific inhibition of PDKs by DCA was reported to improve tumor response to several cytotoxic agents [25]. This synergistic effect of DCA was ascribed to an enhanced ROS production that in turn may cause a higher sensitivity to cell death signals [14]. Our study confirmed that downregulation of PDK1 enhances tumor response to TKI treatment in oncogene-driven NSCLC cell lines.

Although a number of evidence indicate that the glycolytic phenotype is associated with the chemo-resistance and radio-resistance of cancer cells, its causative roles in the development of resistant tumors and the mechanisms underlying such development have not been completely elucidated [26,27]. Multiple molecular mechanisms may cause drug resistance in cancer cells including an enhanced cell survival to apoptotic stimuli. Our study showed that PDK1 may serve as a key molecule linking the glycolytic phenotype to chemo-resistance and radio-resistance. Oncogene and/or HIF-1α-dependent upregulation of PDK1 promotes glycolysis through the inactivation of the PDHC, and upregulated PDK1 simultaneously interacts with anti-apoptotic proteins on the surface of mitochondrial membrane, thus promoting resistance to apoptotic stimuli (Figure 6C, left panel). In addition to its canonical location in the mitochondrial matrix, PDK1 has been detected also in the outer mitochondrial membrane where it can be phosphorylated and activated by several oncogenic tyrosine kinases [28]. Our findings indicate that an additional role of PDK1 on the surface of mitochondrial membrane is the formation of macromolecular complexes with anti-apoptotic proteins.

In the absence of PDK1 (Figure 6C, right panel), the levels of Bcl-2/Bcl-xL in the mitochondrial fractions were reduced, but their destiny is still unknown. They can be either redistributed in the ER or degraded. In this respect, previous studies reported that PKM2 can translocate to mitochondria under oxidative stress in glioblastoma cell lines [29]. Mitochondrial PKM2 was shown to interact with and phosphorylate Bcl-2, thus preventing its degradation. Furthermore, the disruption of PKM2 interaction with Bcl-2 promoted its proteosome-mediated degradation. In our study, the interaction between PKM2 and Bcl-2/Bcl-xL at the ER–mitochondria interface appeared to be dependent from PDK1.

## 4. Materials and Methods

### 4.1. Cell Lines and Treatment

Two NSCLC cell lines were obtained from and authenticated by the American Type Culture Collection. In particular, H1993 cells are reported to have a high level of *MET* gene amplification (15 copy numbers) [30,31] and wild type EGFR [32]. H1975 cells bear an activating point mutation in exon 21 (L858R) of the kinase domain of EGFR, which also harbors the T790M mutation, conferring resistance upon erlotinib [33,34]. The cell lines used were periodically tested negative for mycoplasma contamination. H1993 and H1975 cells were grown in RPMI medium supplemented with 10% fetal bovine serum, 100 IU/mL penicillin and 50 μg/mL streptomycin in a humidified incubator with 5% CO_2_ at 37 °C.

The cells were treated with inhibitors of double mutant EGFR^L858R/T790M^ such as WZ4002 (1 μM, S1173, Selleck Chemicals, Houston, TX, USA), MET inhibitors including PHA-665,752 (1 µM, S1070, Selleck Chemicals) and crizotinib (1 µM, S1068, Selleck Chemicals) or vehicle for 48 h [20].

Annexin V assays were performed by using a commercially available kit (BD Biosciences, NJ, USA), as previously described [35]. Briefly, shCTRL and shPDK1 H1975 cells (1 × 10^6^ cells/sample, exposed or not to 1 μM WZ4002 for 24 h, were incubated with 5 µL Annexin V-FITC diluted in 200 µL binding buffer for 10 min at room temperature in the dark. Then, the cells were washed with binding buffer, incubated with 10 µL propidium iodide (20 µg/mL) in 190 µL binding buffer and immediately analyzed using a BD FACSAria II flow cytometer (BD Biosciences, Franklin Lakes, NJ, USA). At least 3 independent experiments were performed in triplicate, and data were pooled.

### 4.2. shRNA and siRNA Interference

PDK1 targeted (shPDK1) and non-targeting (shCTRL) short hairpin RNA were purchased from Sigma-Aldrich (product number TRCN0000006263 and SHC002, respectively). In H1993 and H1975 cells, PDK1 knockdown was achieved using lentiviral vectors (MISSION Lentiviral Packaging Mix, SHP001, Sigma-Aldrich Saint Louis, MO, USA). Lentiviral vectors expressing either a PDK1 targeted shRNA (shPDK1) and non- targeting shRNA (shCTRL) were produced transfecting 293 T cell line according to manufacturer’s instructions. Stable transfected cells were selected with puromycin (3 ng/mL, ANT-PR-1, InvivoGen, Toulouse, France) for 10 to 14 days. Antibiotic resistant colonies were pooled and expanded for further analysis.

In selected experiments, cells were transfected with siRNA targeting PKM2 (sense 5′CCAUAAUCGUCCUCACCAAUU3′, antisense 3′UUGGUAUUAGCAGGAGUGGUU5′), PDK1 (L-005019-00-0005) or LDH-A (L-008201-00-0005). Targeted siRNA pools (ON-TARGETplus SMARTpool) and control non-targeting siRNA pool (siCTRL, FE5D0018101005) were purchased from Dharmacon, Inc (Lafayette, CO, USA) and used according to the manufacturer’s instructions. Briefly, H1993 and H1975 cells were plated and allowed to attach for 24 h. Then, cells were transfected with 100 nM siRNAs using the Dharmafect reagent (T-2001-02, Dharmacon), and after 24 h EGFR or MET inhibitors were added for further 48 h.

### 4.3. Immunoblotting Analysis and Immunoprecipitation

Whole cell lysates were prepared as previously described [20]. Briefly, untreated and treated cells were lysed on ice in RIPA lysis buffer (R0278, Sigma-Aldrich) with protease (P8340, Sigma-Aldrich) and phosphatase inhibitors (P0044, Sigma-Aldrich) and kept on ice for 30 min. The suspension was homogenized and centrifuged at 13000× *g* at 4 °C for 20 min. Mitochondrial fractions were prepared as previously described [36]. Briefly, cells were lysed and homogenized in an ice-cold buffer (225 mM mannitol, 75 mmol/L sucrose, 0.1 mM EGTA and 30 mM Tris-HCl pH 7.4) using a Dounce homogenizer and then centrifuged at 600× *g* for 5 min at 4 °C. The supernatant was centrifuged at 7000× *g* for 10 min at 4 °C, and the pellet obtained was washed in another ice-cold buffer (225 mM mannitol, 75 mM sucrose and 30 mM Tris-HCl pH 7.4). After a centrifugation at 10,000× *g* for 10 min at 4 °C, the pellet was resuspended in mitochondria resuspending buffer (250 mM mannitol, 5 mM HEPES pH 7.4 and 0.5 mM EDTA).

Western blot analysis of proteins from different lysates was performed by using a standard procedure. Antibodies used for Western blotting included mouse monoclonal antibodies against IP3R3 (610313, BD Biosciences), HIF-1α (610958, BD Biosciences), Bcl-xL (sc-8392, Santa Cruz Biotechnology, Dallas, TX, USA), Bcl-2 (sc-509, Santa Cruz Biotechnology), p53 (sc-126, Santa Cruz Biotechnology), α-tubulin (T9026, Sigma-Aldrich), actin (A4700, Sigma-Aldrich), cytochrome C (556433, BD Biosciences) and PDH-E1α (ab110416, Abcam, Cambridge, UK). A cocktail of 5 mAbs against OXPHOS (ab110411, Abcam) recognized the following proteins: 20 kD subunit of Complex I (20 kD), COX II of Complex IV (22 kD), 30 kD Ip subunit of Complex II (30 kD), core 2 of Complex III (~50 kD) and F1α (ATP synthase) of Complex V (~60 kD); rabbit monoclonal antibodies against vinculin (4650, Cell Signaling Technology, Danvers, MA, USA), PDHK1 (3820, Cell Signaling Technology), PKM2 (4053, Cell Signaling Technology), Hexokinase II (2867, Cell Signaling Technology), LDH-A (3582, Cell Signaling Technology), p-BAD^Ser112^ (5284, Cell Signaling Technology), cleaved caspase 3 (9661, Cell Signaling Technology) and BAX (5023, Cell Signaling Technology); rabbit polyclonal antibody p-PKM2^Tyr105^ (3827, Cell Signaling Technology), lamin a/c (2032, Cell Signaling Technology), cyclin D1 (2922, Cell Signaling Technology), p-PDH^Ser293^ (ab92696, Abcam), p-PKM2^Ser37^ (11456, Signalway Antibody, College Park, MD, USA), VDAC1/porin (ab15895, Abcam) and BIM (559685, BD Biosciences). A commercially available ECL kit (Advansta, San Jose, CA, USA) was used to reveal the reaction.

Immunoprecipitation was performed as previously described [37]. Briefly, precleared proteins from cell lysates were incubated overnight at 4 °C with anti-PDK1, anti-PKM2, anti-IP3R3, anti-Bcl-2 and anti-Bcl-xL antibodies. The immunoprecipitated proteins recovered by absorption to EZview Red Protein A Affinity gel (P6486, Sigma-Aldrich) were separated by SDS-PAGE, transferred to PVDF membranes and probed for the indicated proteins.

### 4.4. Glucose Consumption, Intracellular ATP and Citrate Levels in Cultured Tumor Cells

In parallel experiments, both cell lines transfected with shCTRL and shPDK1 were analyzed for glucose levels in the conditioned media and for intracellular ATP concentrations in basal conditions and in response to TKIs. Briefly, conditioned media were removed, centrifuged at 13000× *g* at 4 °C for 10 min and then assayed for glucose concentrations using the Glucose Assay Kit (GAG020-1KT, Sigma-Aldrich), following manufacturer’s instructions. Intracellular ATP determination was performed using the ATPlite Luminescence Assay (6016941, Perkin Elmer; Waltham, MA, USA) following manufacturer’s instructions. Briefly, cells were lysed, incubated with the ATP reaction mixture for 5 min and then subjected to luminescence measurements. Moreover, citrate levels were determined in shCTRL and shPDK1 cells using the Citrate Assay Kit (Sigma-Aldrich), following manufacturer’s instructions. Absolute glucose, ATP and citrate levels were calculated from the corresponding standard curve and normalized to 10^6^ cells. At least three independent experiments were performed, and data were pooled.

### 4.5. Oxygen Consumption and Extracellular Acidification Rates

The oxygen consumption rate (OCR) and extracellular acidification rate (ECAR) were determined by using the Seahorse Extracellular Flux Analyzer (XF-96, Seahorse Bioscience, North Billerica, MA, USA) for both cell lines. OCR was measured in basal conditions and after the subsequent addition of 5 μM oligomycin, 1.5 μM carbonylcyanide-4-(trifluoromethoxy)-phenylhydrazone (FCCP) and 1 μM rotenone/antimycin A. The ECAR was simultaneously measured in basal conditions and after the subsequent addition of 10 mM glucose, 5 μM oligomycin and 100 mM 2-deoxyglucose. OCR and ECAR were determined in quintuplicate samples of untreated and treated cells, and data were expressed as pmol/min and mpH/min, respectively.

### 4.6. PDK1 Knockdown and Tumor Growth in Animal Models

Female BALB/c (nu/nu) mice that were 6 weeks old and weighing 15–20 g were purchased from the Charles River Laboratories (Milan, Italy). All animal experimental procedures were approved by the Institutional Review Board and by the Italian Ministry of Health-Animal Welfare Direction (Protocol No. 324/2017-PR 18-04-2017). Animals were randomized in two groups. H1993 cells (5–10 × 10^6^) stably transfected with shCTRL and shPDK1 were resuspended in 200 μL RPMI medium and injected s.c. into the flank of nude mice of each group. Cells were then allowed to grow, and the diameter of each tumor was measured daily. When tumor volume reached approximately 100 mm^3^, animals were sacrificed. Then, tumors were surgically removed, immediately frozen in liquid nitrogen and stored at −80 °C until used. Tumor samples were homogenized on ice in RIPA lysis buffer with protease and phosphatase inhibitors (Sigma-Aldrich) by using a Dounce homogenizer followed by passages through a 26-gauge needle. The suspension was clarified by centrifugation at 13,000× *g* for 30 min at 4 °C and tested for the expression of several proteins by Western blot analysis.

### 4.7. Statistical Analysis

Statistical analysis was performed using the software MedCalc for Windows, version 12.7.0.0, (MedCalc Software, Mariakerke, Belgium). The unpaired Student’s *t*-test was used when appropriate for comparing means. Differences between means were considered statistically significant for *p* < 0.05.

## 5. Conclusions

In conclusion, our study highlighted a non-canonical role of PDK1 as a negative regulator of apoptosis through the formation of macromolecular complexes at the ER–mitochondria interface in oncogene-driven NSCLC. Co-targeting of PDK1 and oncogene driver in NSCLC strongly enhanced TKI effects on glucose metabolism and apoptosis, thus providing a rational basis for combined therapeutic strategies in NSCLC patients.

## Data Availability

The data presented in this study are available in the article and in the Supplementary Materials.

## Supplementary Materials

The following are available online at https://www.mdpi.com/article/10.3390/cancers13164133/s1, Figure S1. Citrate levels were determined in shCTRL and shPDK1 H1975 cells and expressed as nmol of citrate normalized to 10^6^ cells, Figure S2. Levels of OXPHOS in whole cell lysates from siCTRL and siPDK1 H1993 cells exposed to 1 µM PHA-665-752 for 48 h, Figure S3. Annexin V assay was performed in shCTRL and shPDK1 H1975 cells exposed or not to WZ4002 for 24 h, Figure S4. Whole blot image from Figure 1A, Figure S5. Whole blot image from Figure 1C, Figure S6. Whole blot image from Figure 1C, Figure S7. Whole blot image from Figure 2A, Figure S8. Whole blot image from Figure 3A, Figure S9. Whole blot image from Figure 3B, Figure S10. Whole blot image from Figure 3C, Figure S11. Whole blot image from Figure 3C, Figure S12. Whole blot image from Figure 3C, Figure S13. Whole blot image from Figure 3C, Figure S14. Whole blot image from Figure 3D, Figure S15. Whole blot image from Figure 4A, Figure S16. Whole blot image from Figure 4A, Figure S17. Whole blot image from Figure 4B, Figure S18. Whole blot image from Figure 4C, Figure S19. Whole blot image from Figure 4D, Figure S20. Whole blot image from Figure 4E, Figure S21. Whole blot image from Figure 4F, Figure S22. Whole blot image from Figure 4G, Figure S23. Whole blot image from Figure 5A, Figure S24. Whole blot image from Figure 5B, Figure S25. Whole blot image from Figure 6B.
