# Supplementary material for: Non-Canonical Role of PDK1 as a Negative Regulator of Apoptosis through Macromolecular Complexes Assembly at the ER–Mitochondria Interface in Oncogene-Driven NSCLC
Source: Cancers (Basel). 2021 Aug 17;13(16):4133. doi: 10.3390/cancers13164133 (PMC8391251; doi:10.3390/cancers13164133)
Supplement: Supplementary file 1 [file cancers-13-04133-s001.zip › cancers-1332075-SI.pdf]

Supplementary Materials: Non-Canonical Role of PDK1 as a Negative Regulator of Apoptosis through Macromolecular Complexes Assembly at the ER–Mitochondria Interface in Oncogene-Driven NSCLC

Viviana De Rosa, Francesca Iommelli, Cristina Terlizzi, Eleonora Leggiero, Rosa Camerlingo, Giovanna G. Altobelli, Rosa Fonti, Lucio Pastore and Silvana Del Vecchio

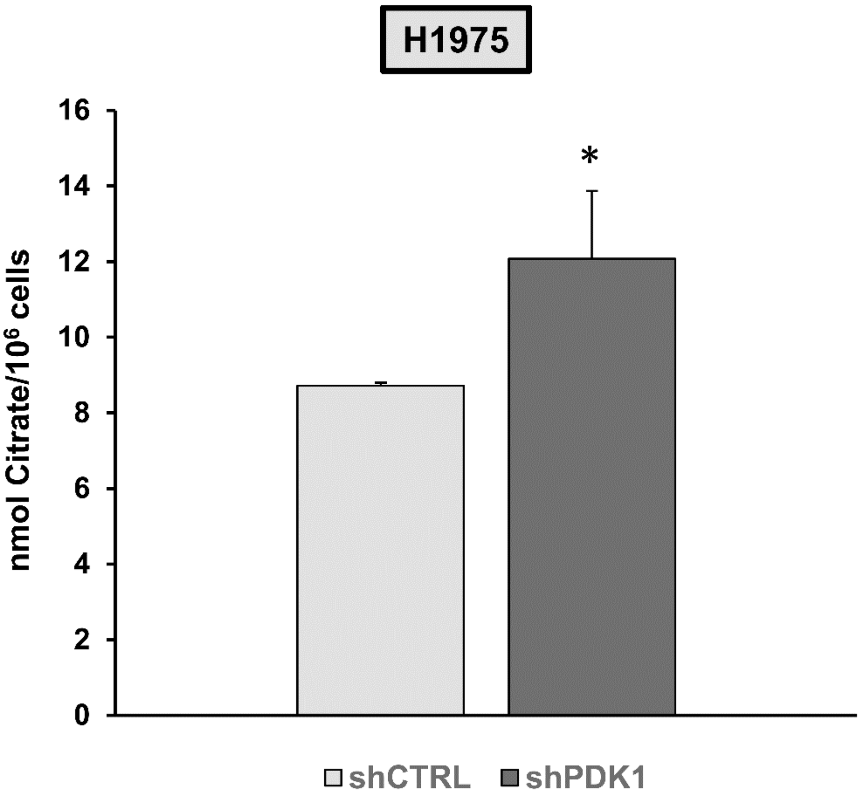

Figure S1. Citrate levels were determined in shCTRL and shPDK1 H1975 cells and expressed as nmol of citrate normalized to 10^6 cells. Data are expressed as mean ± SD. The symbol * indicates statistically significant differences versus untreated shCTRL cells with * $p < 0.05$.

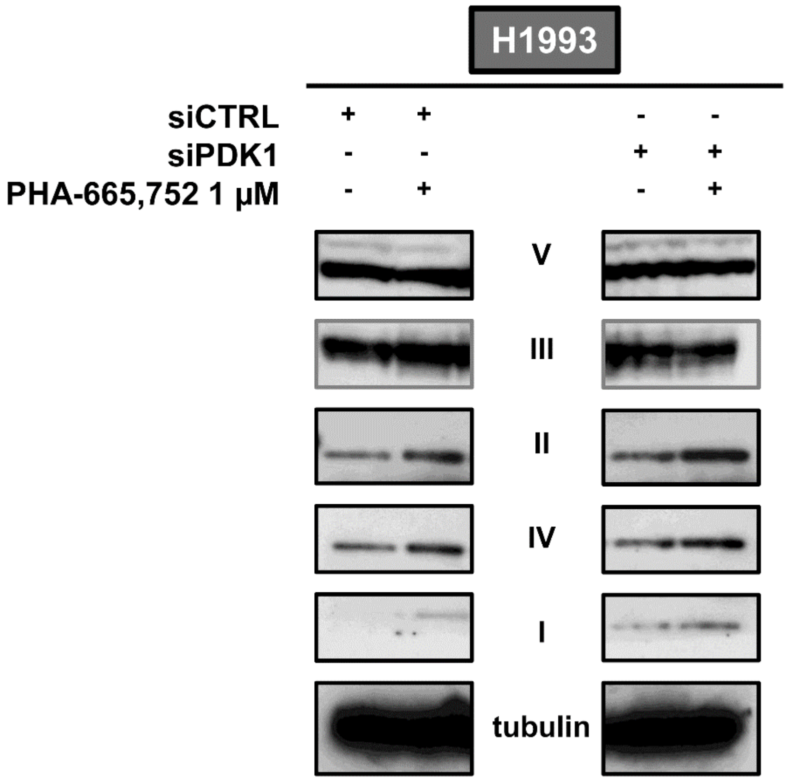

Figure S2. Levels of OXPHOS in whole cell lysates from siCTRL and siPDK1 H1993 cells exposed to 1 μM PHA-665-752 for 48 h. Tubulin served to ensure equal loading.

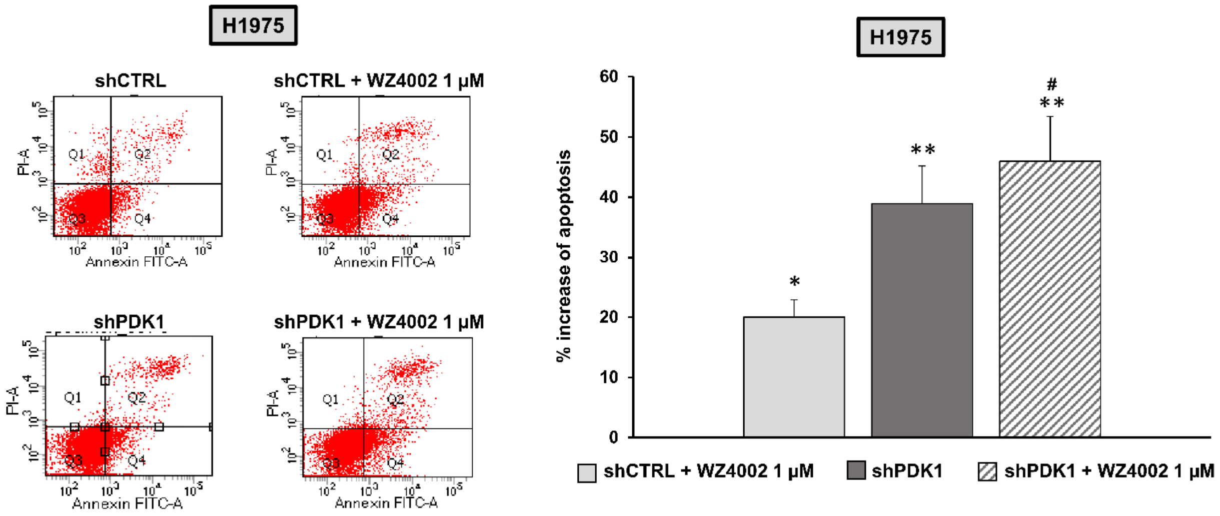

Figure S3. Annexin V assay was performed in shCTRL and shPDK1 H1975 cells exposed or not to WZ4002 for 24 h. Results of a representative assay are shown on the left. The graph on the right shows pooled data expressed as percentage increase of apoptotic cells (mean ± SD) considering untreated shCTRL cells as reference. The symbol * indicates statistically significant differences versus untreated shCTRL cells with * $p < 0.05$ and ** $p < 0.01$; the symbol # indicates statistically significant differences versus treated shCTRL cells with # $p < 0.05$.

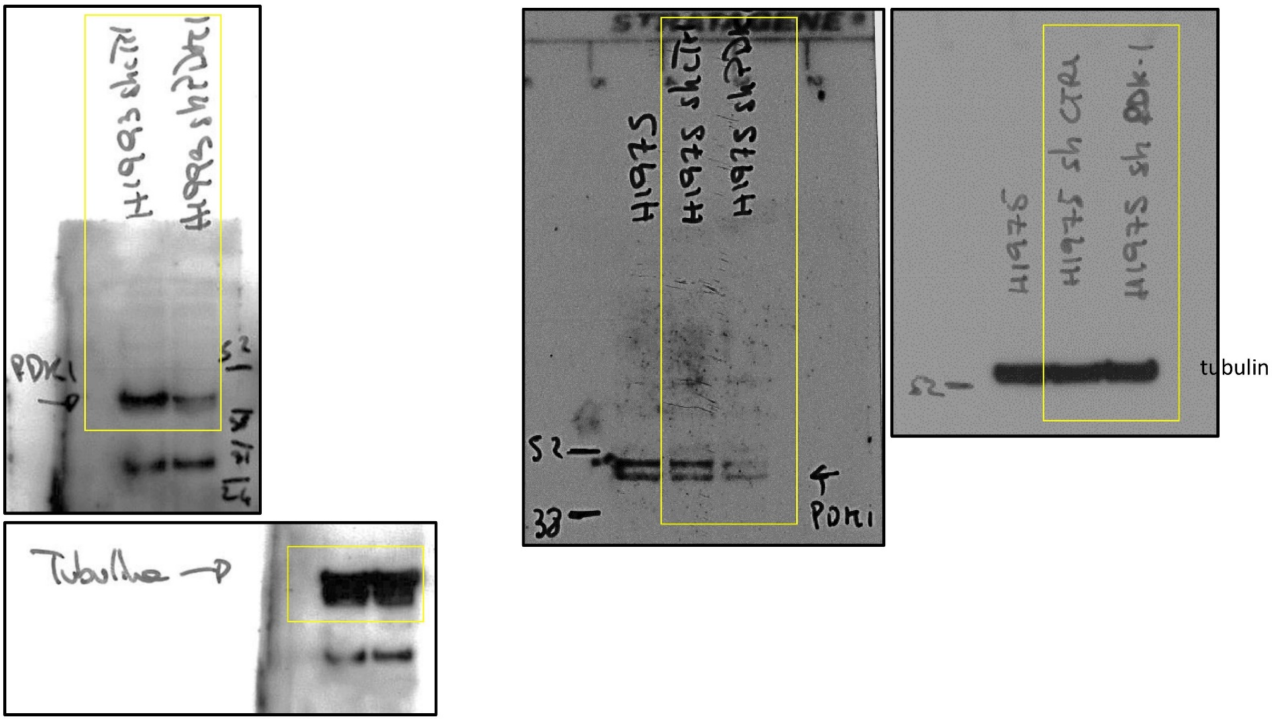

Figure S4. Whole blot image from Figure 1A.

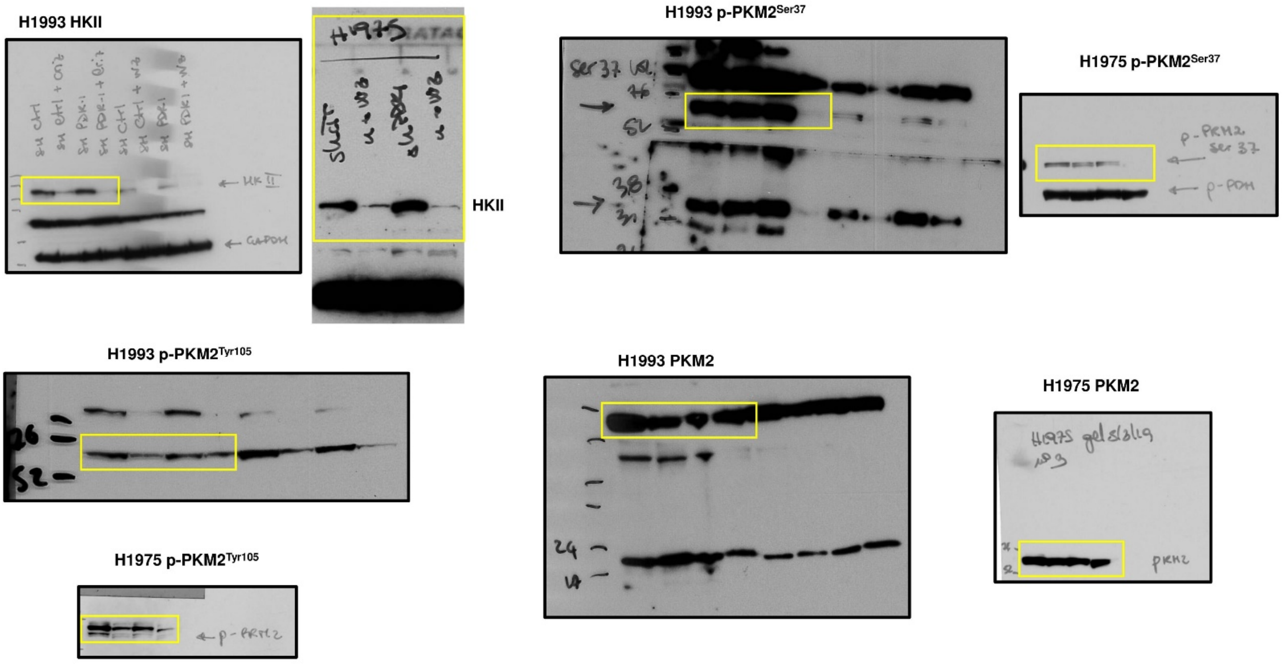

Figure S5. Whole blot image from Figure 1C.

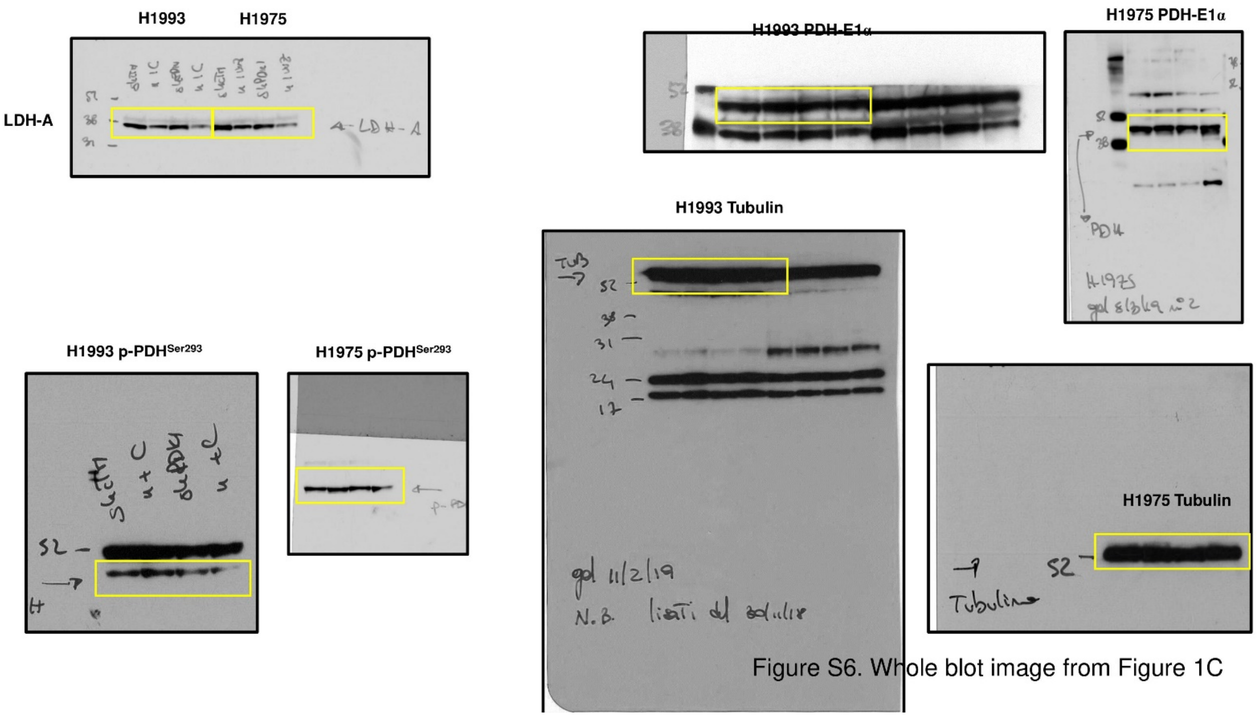

Figure S6. Whole blot image from Figure 1C.

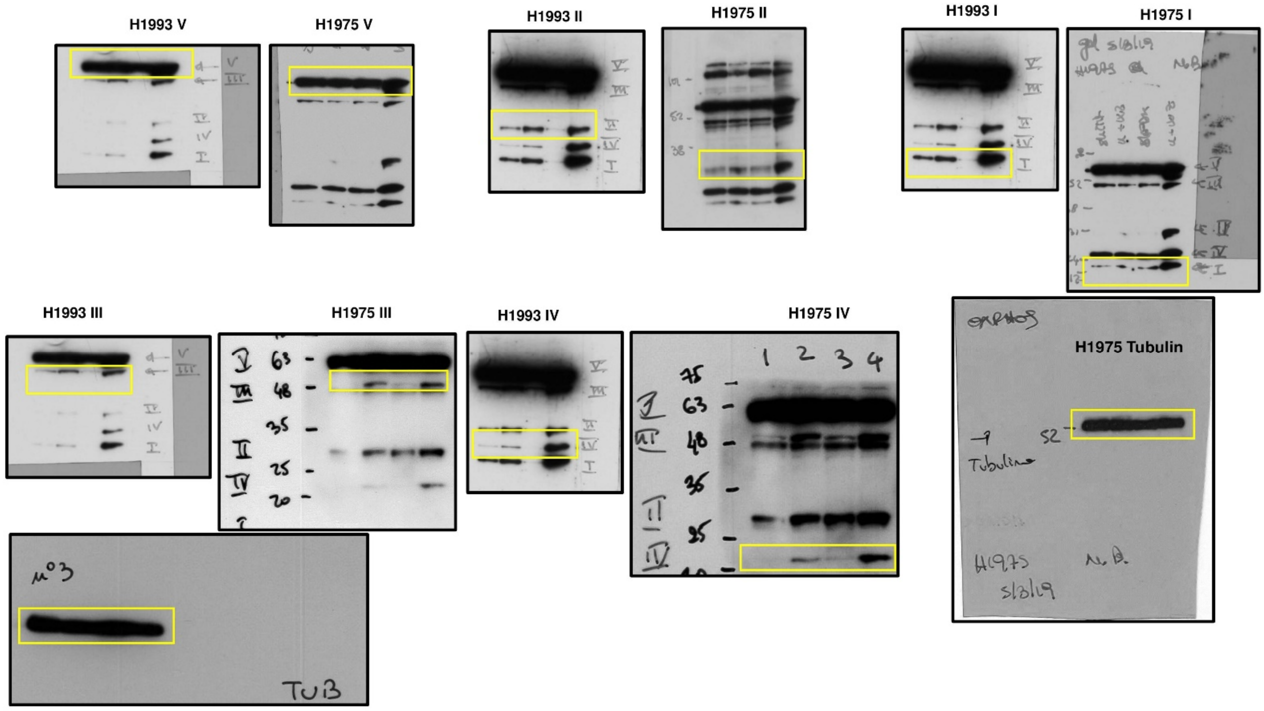

Figure S7. Whole blot image from Figure 2A.

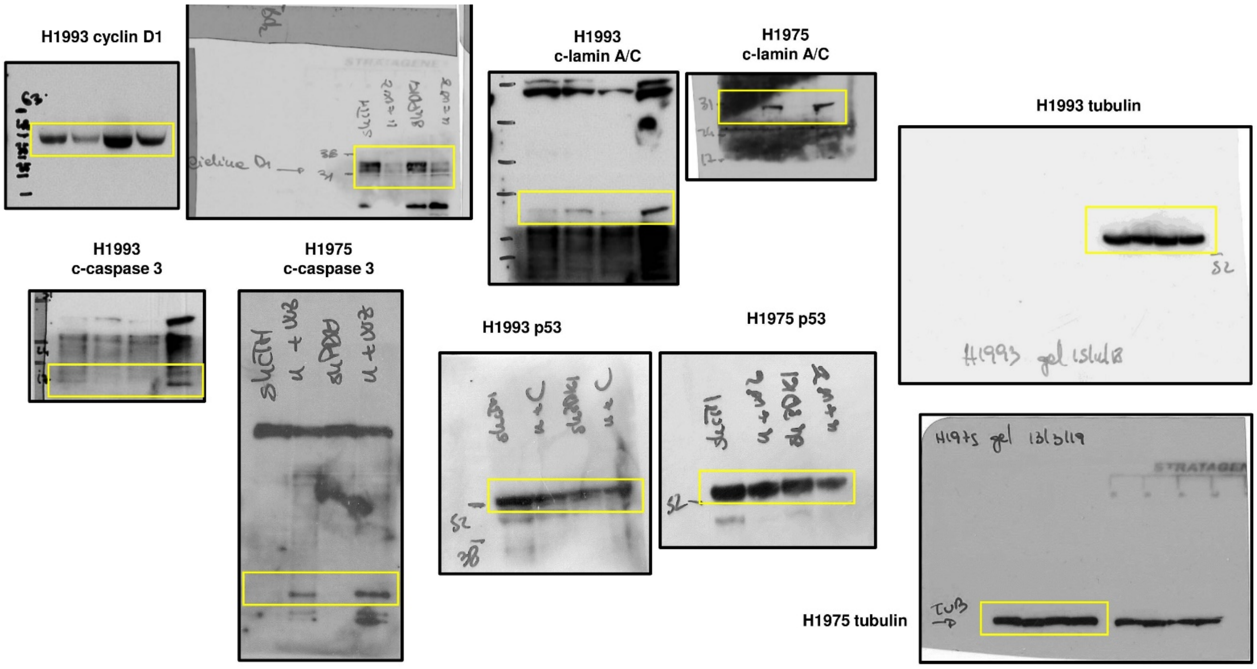

Figure S8. Whole blot image from Figure 3A.

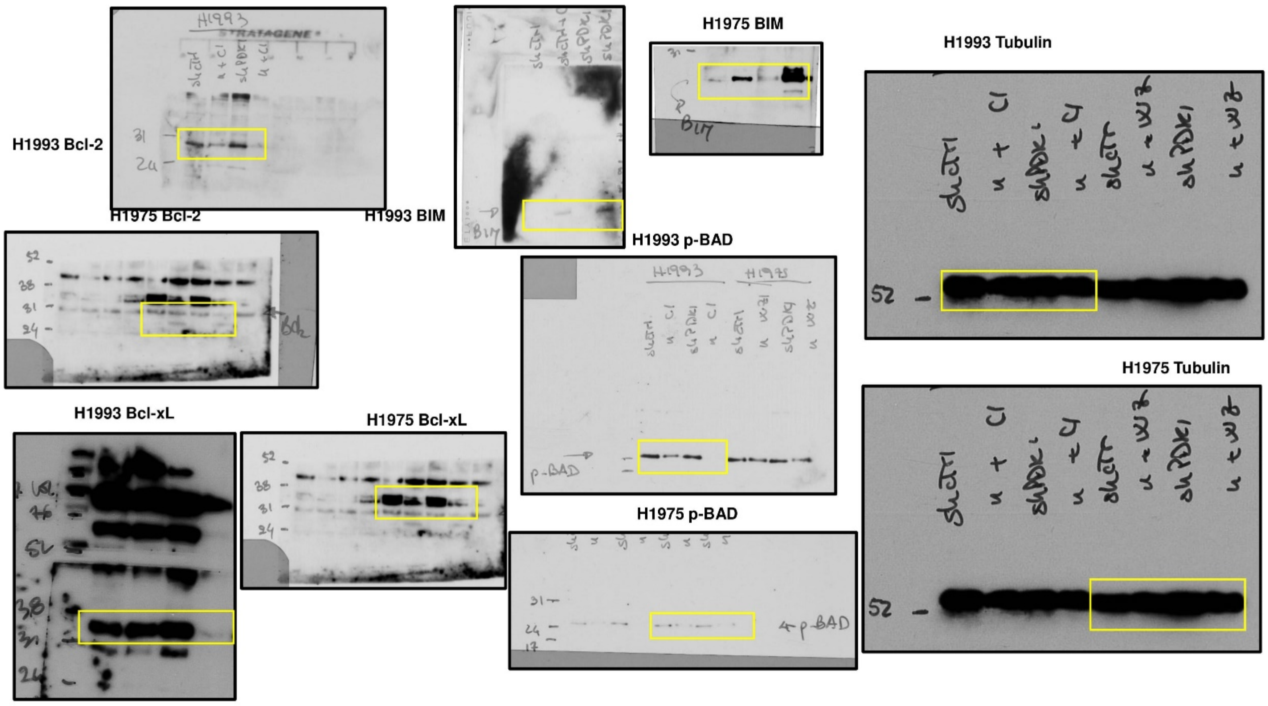

Figure S9. Whole blot image from Figure 3B.

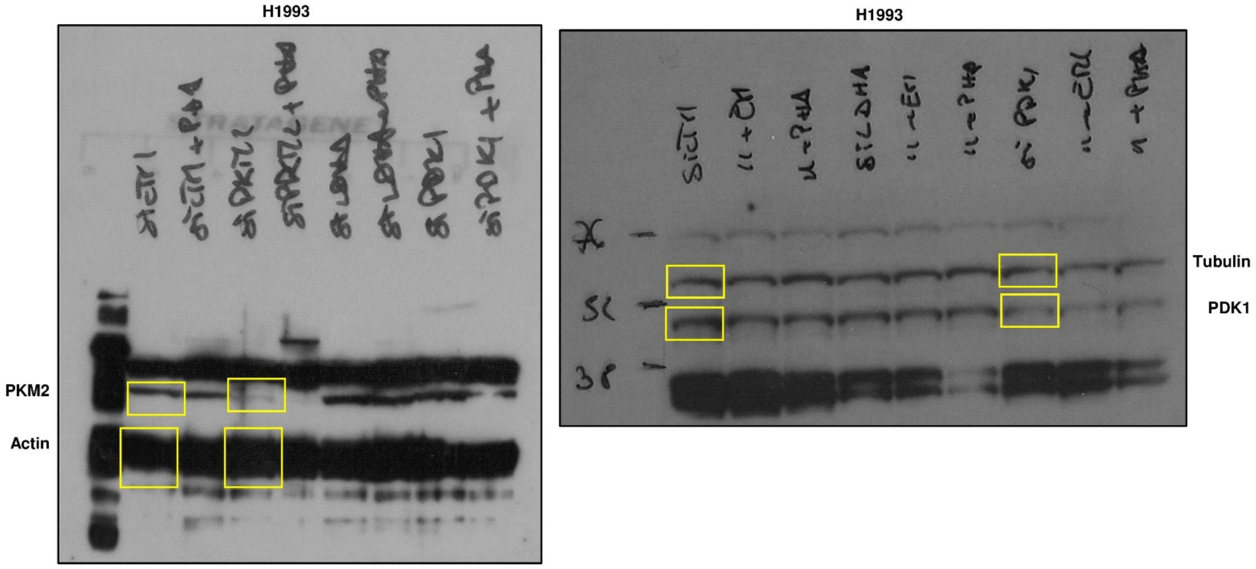

Figure S10. Whole blot image from Figure 3C.

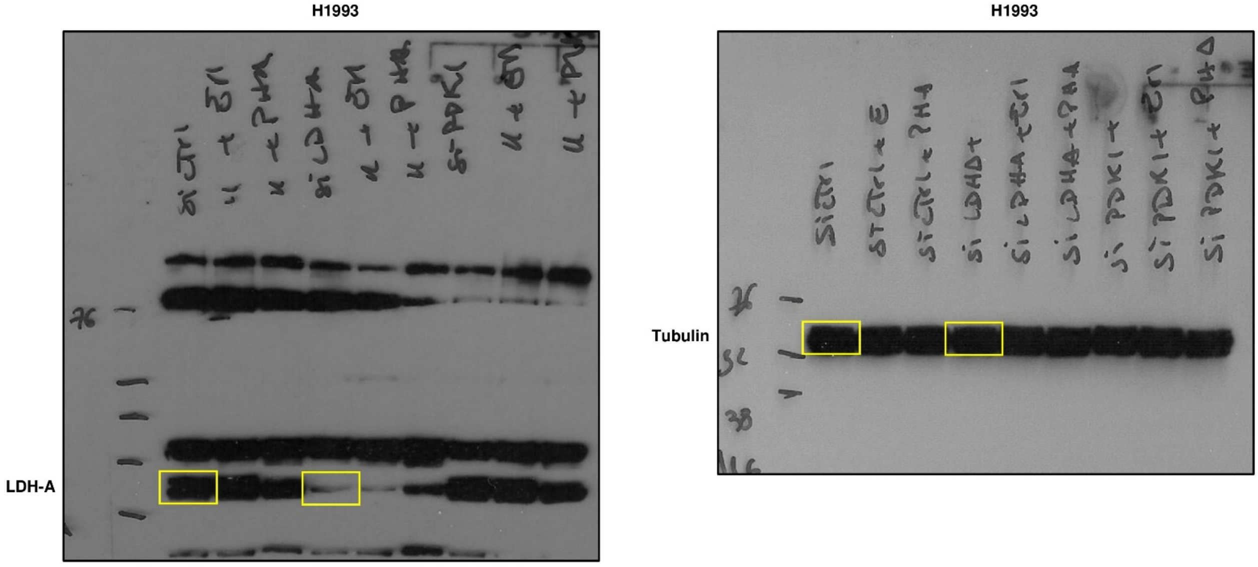

Figure S11. Whole blot image from Figure 3C.

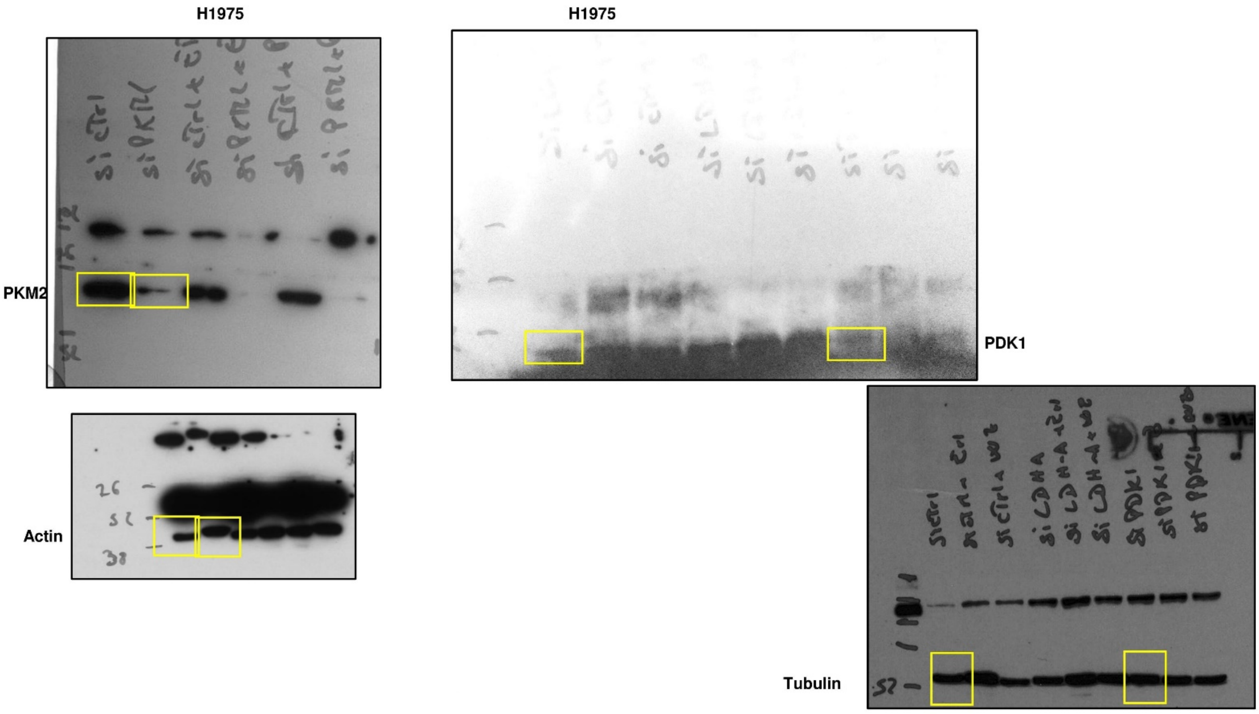

Figure S12. Whole blot image from Figure 3C.

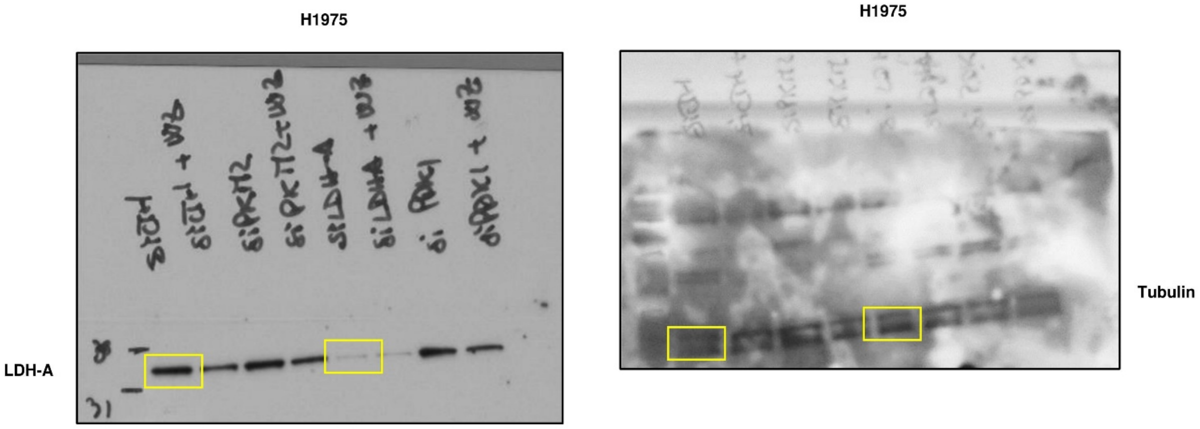

Figure S13. Whole blot image from Figure 3C.

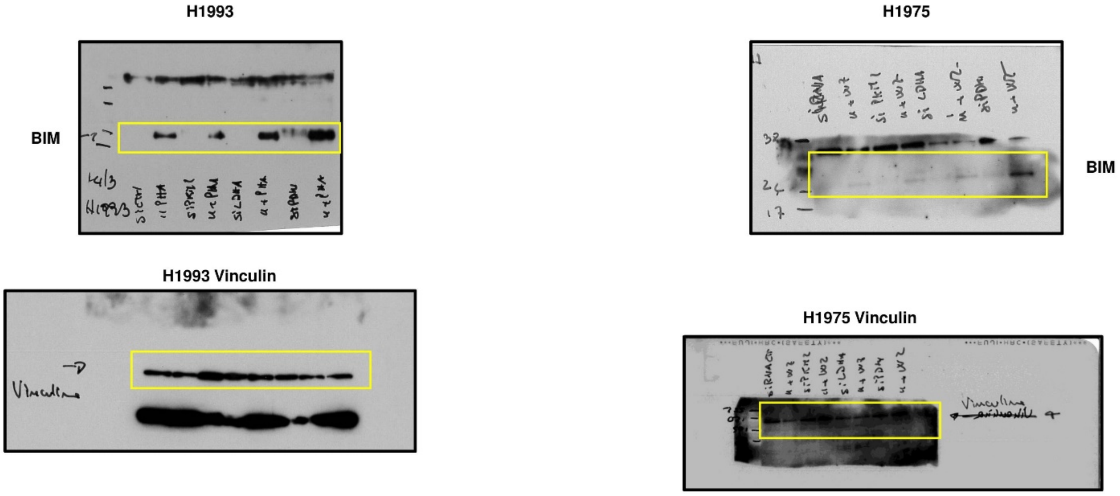

Figure S14. Whole blot image from Figure 3D.

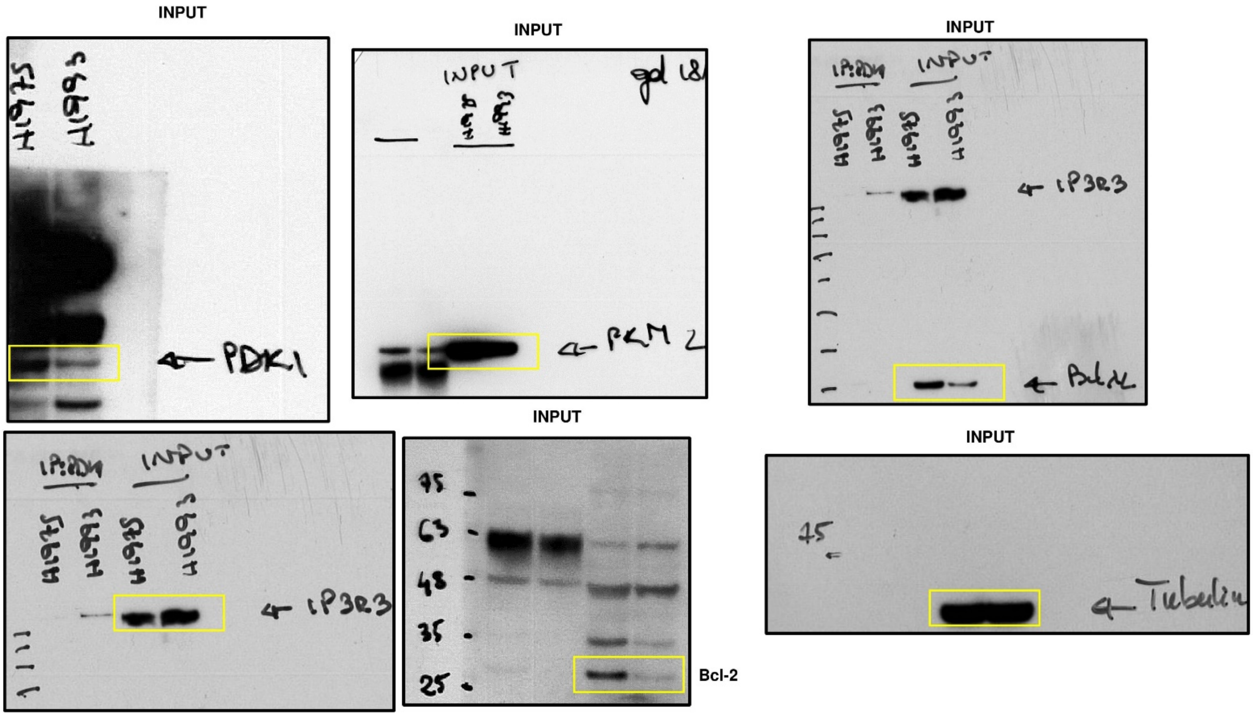

Figure S15. Whole blot image from Figure 4A.

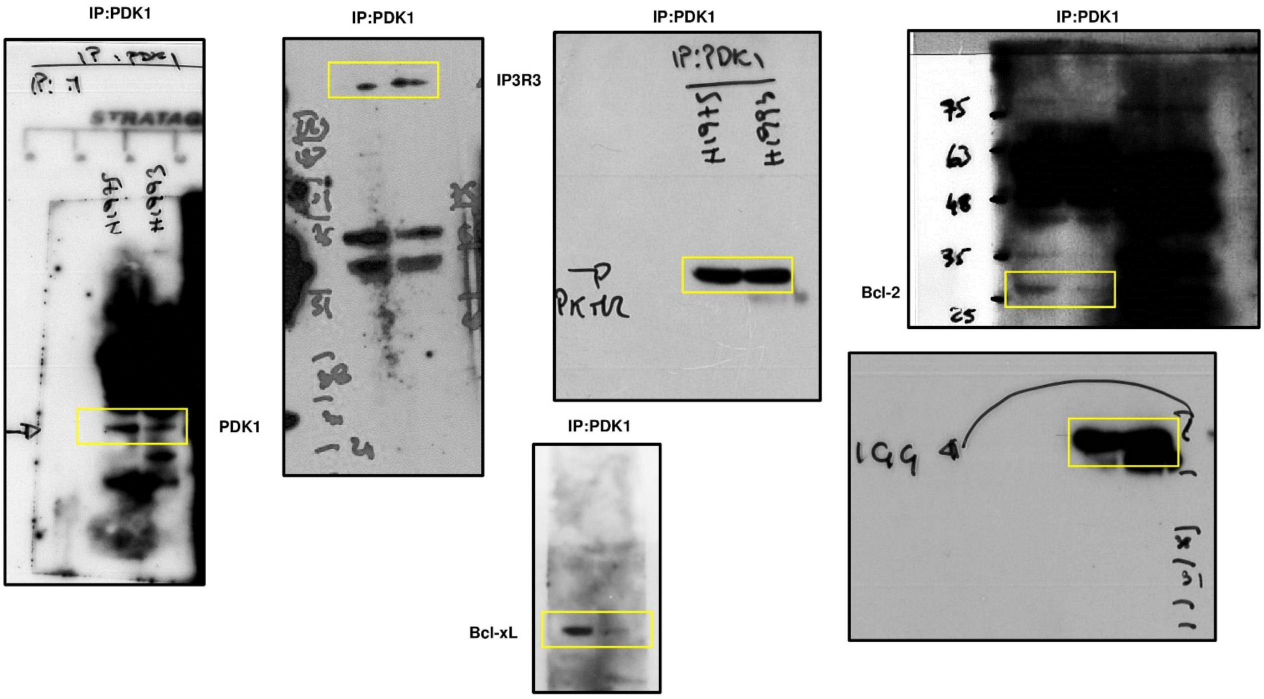

Figure S16. Whole blot image from Figure 4A.

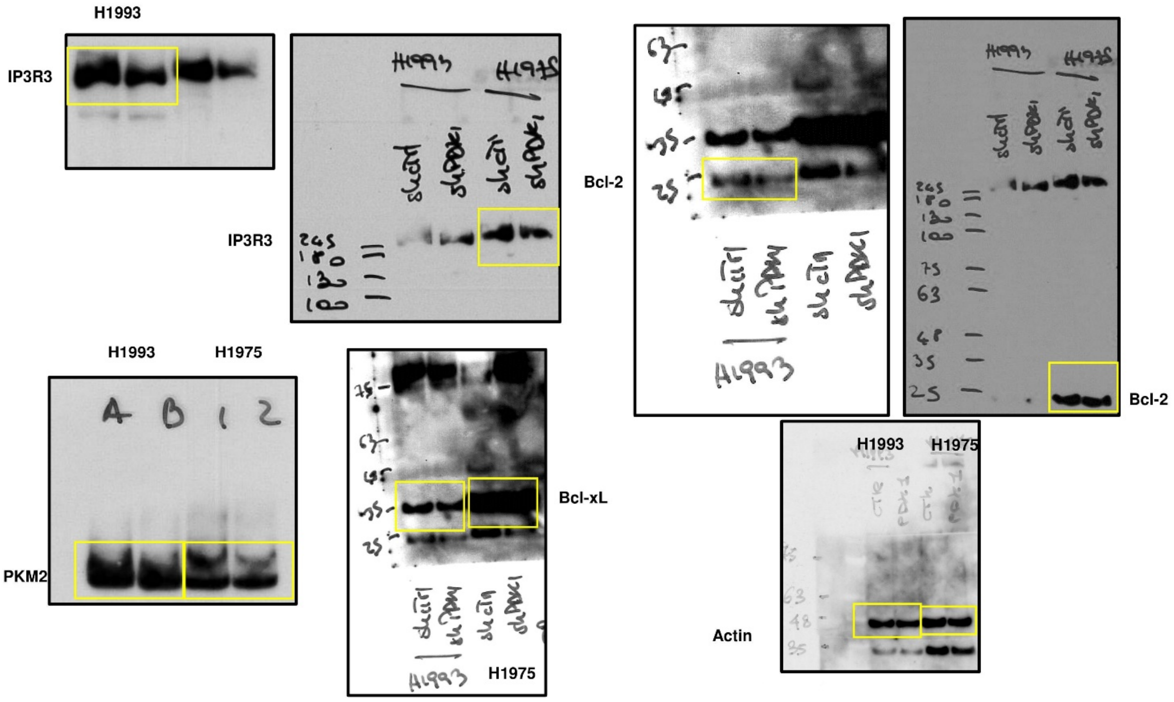

Figure S17. Whole blot image from Figure 4B.

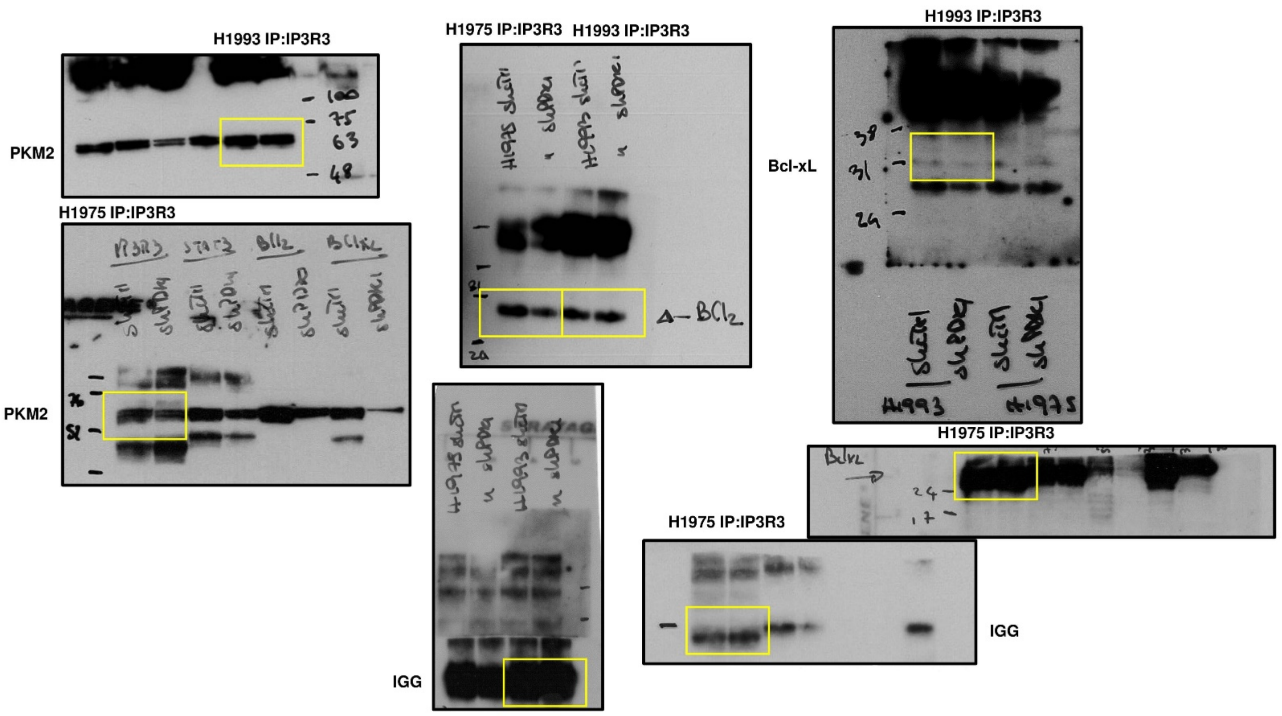

Figure S18. Whole blot image from Figure 4C.

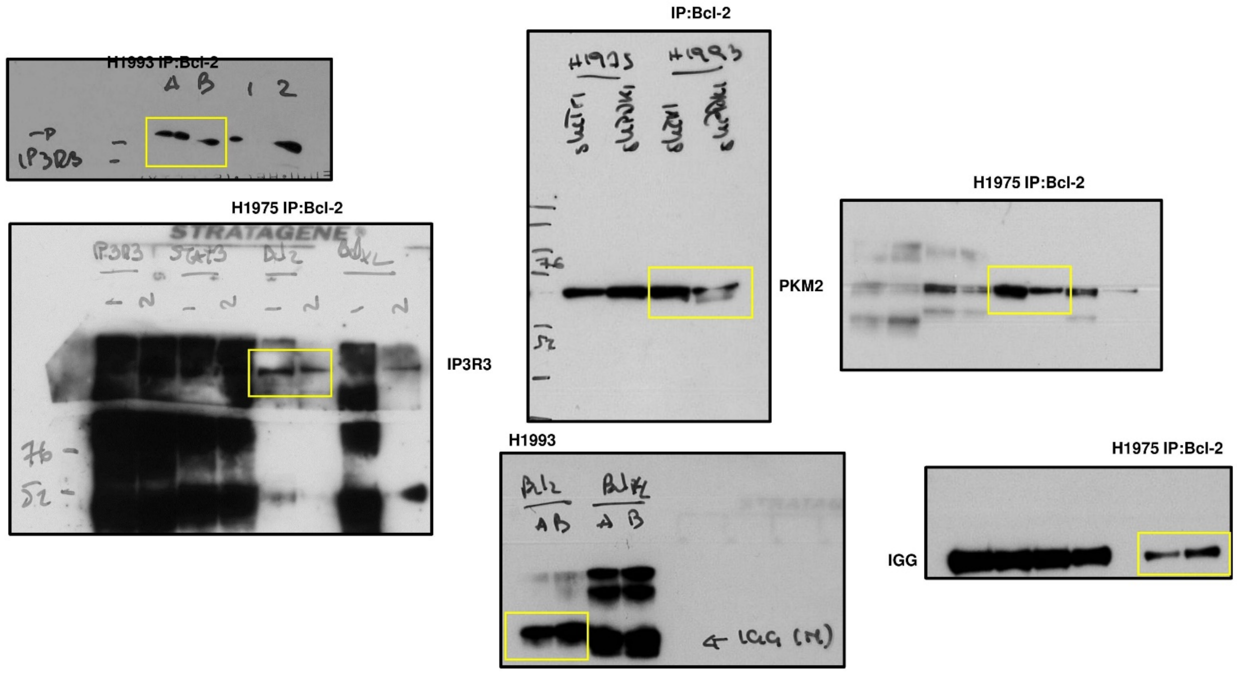

Figure S19. Whole blot image from Figure 4D.

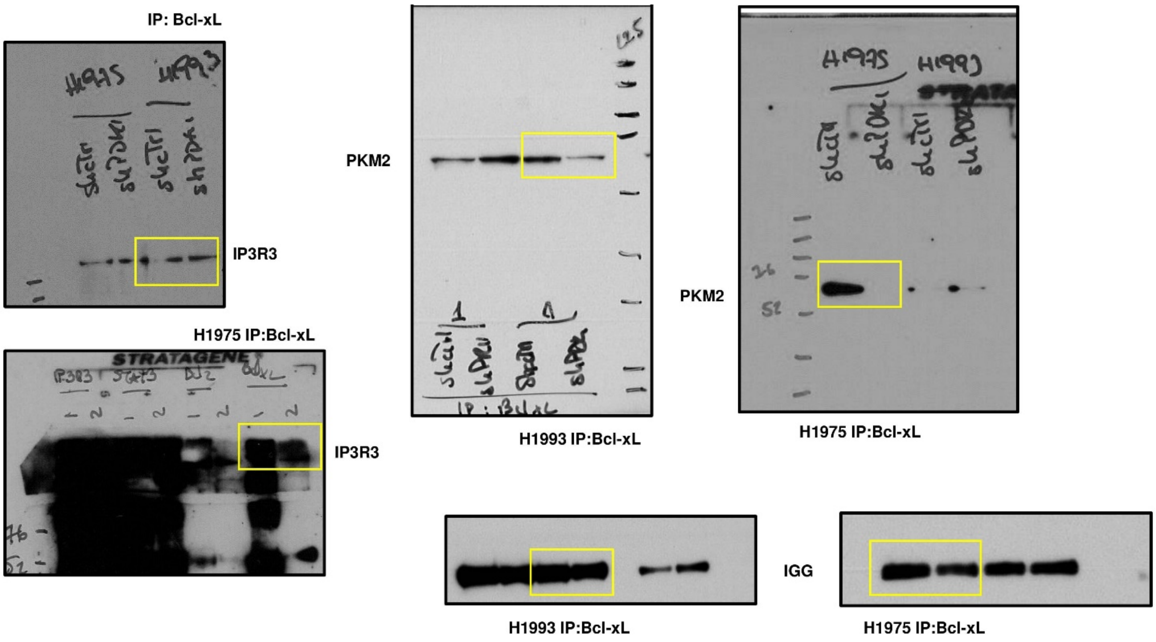

Figure S20. Whole blot image from Figure 4E.

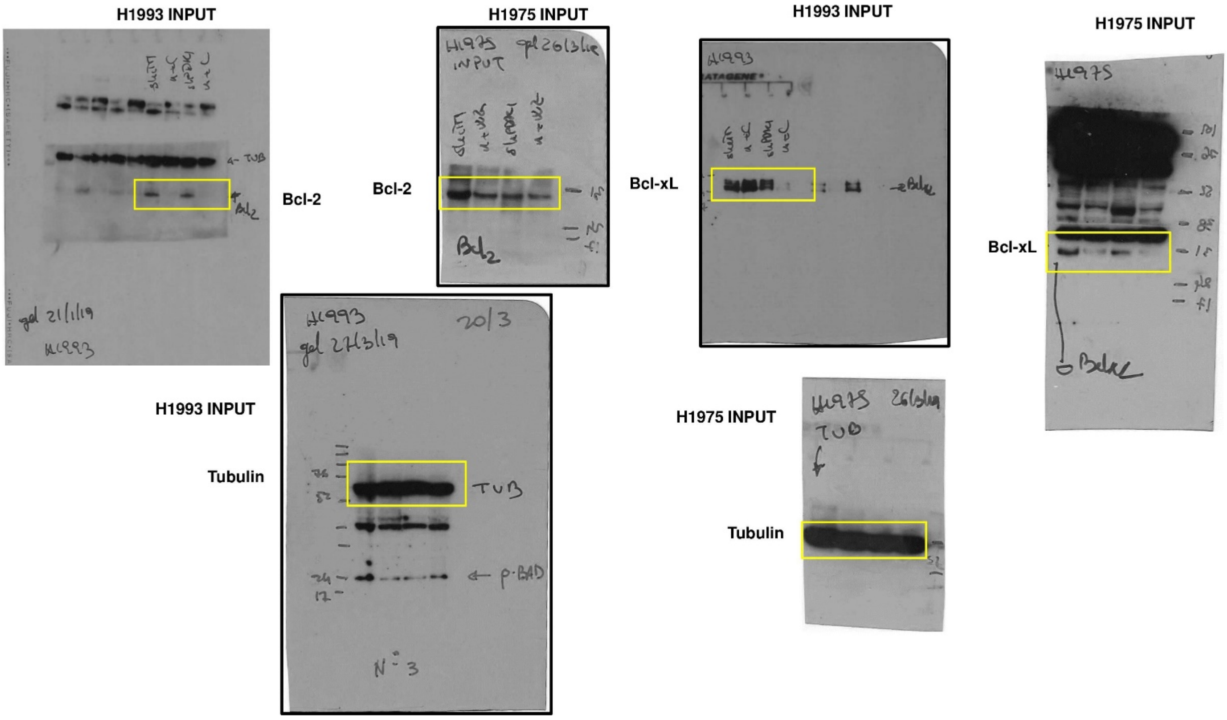

Figure S21. Whole blot image from Figure 4F.

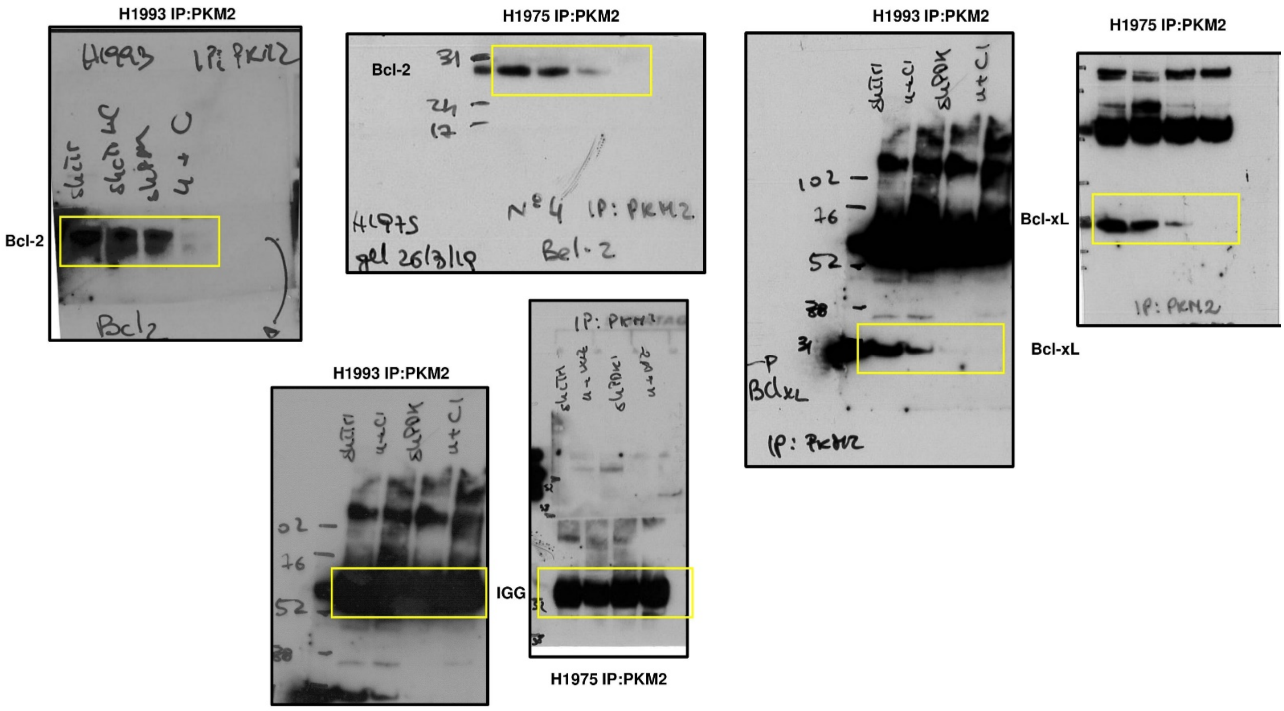

Figure S22. Whole blot image from Figure 4G.

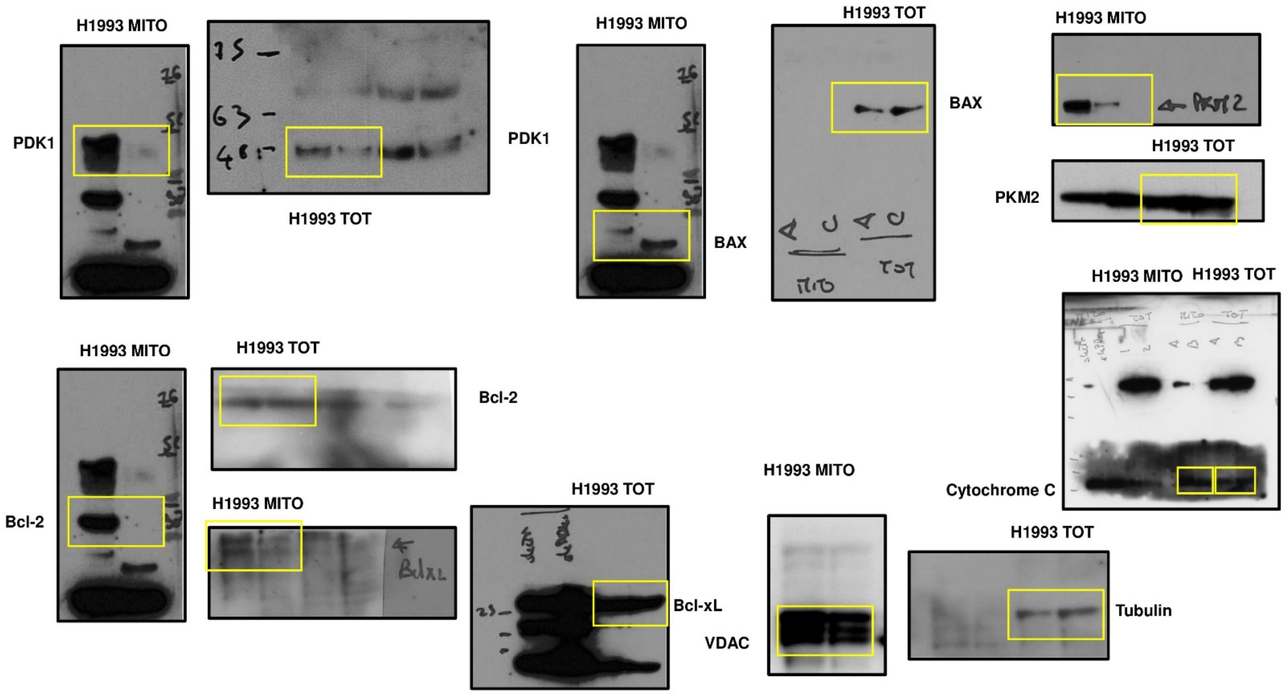

Figure S23. Whole blot image from Figure 5A.

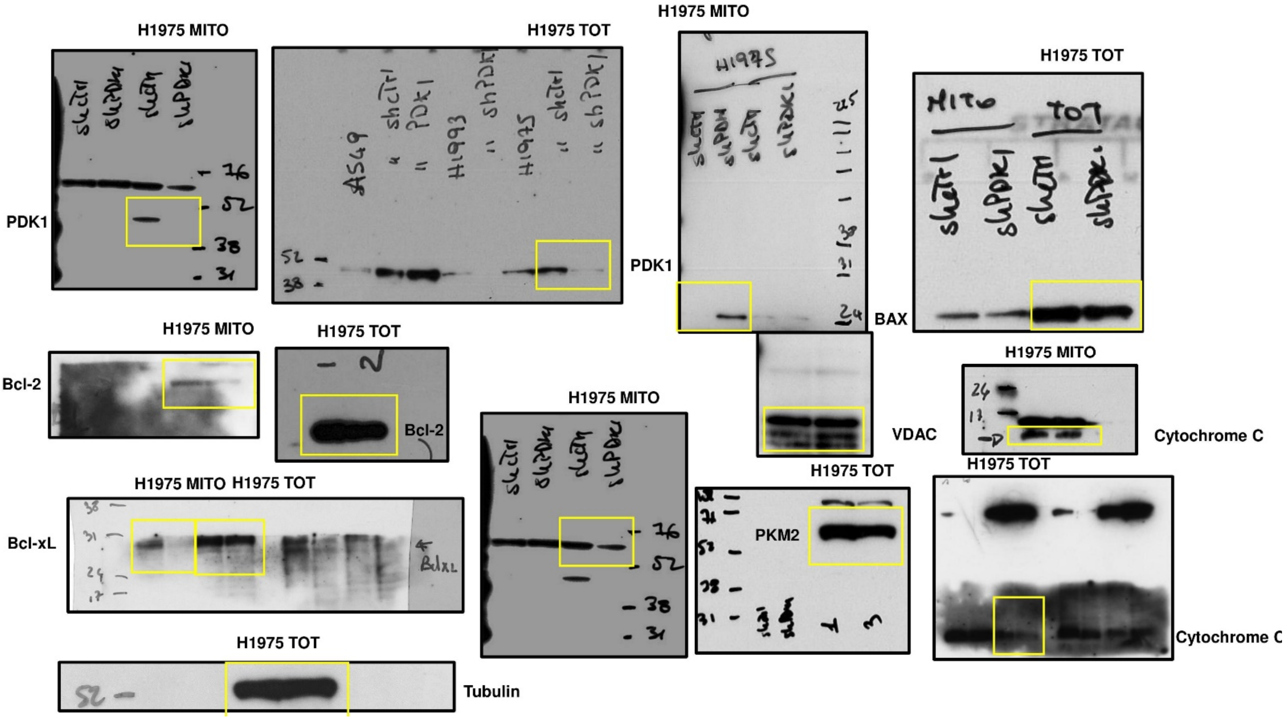

Figure S24. Whole blot image from Figure 5B.

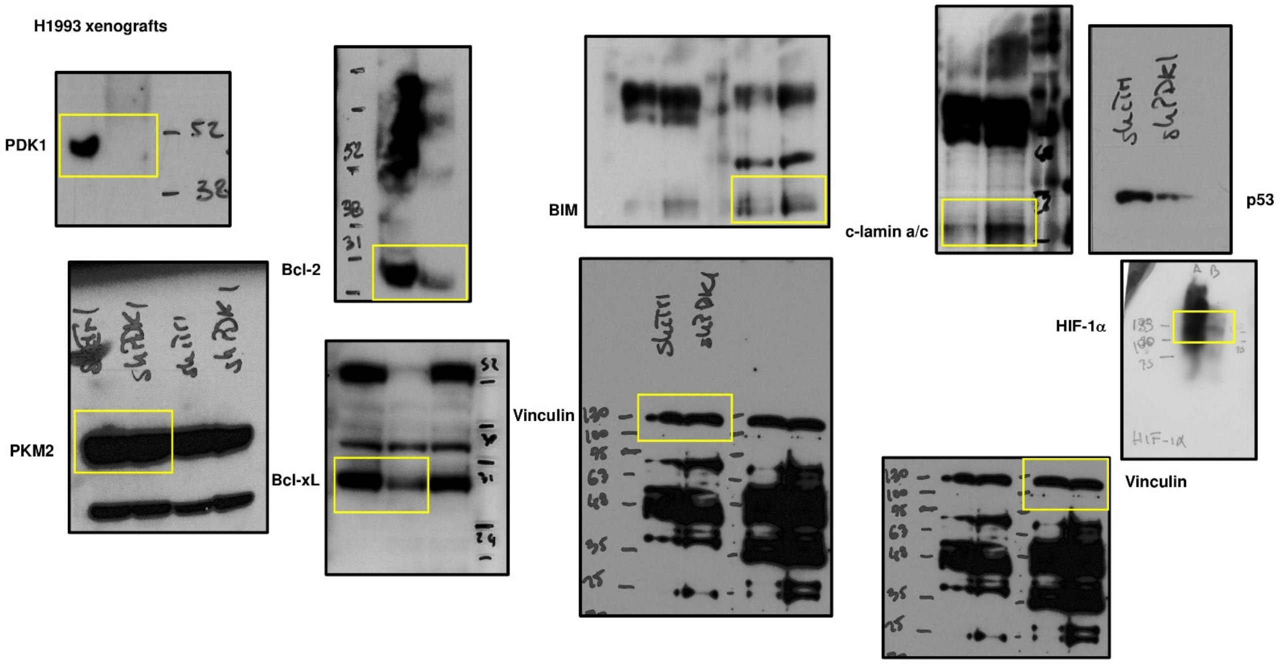

Figure S25. Whole blot image from Figure 6B.
